# Unanchored ubiquitin chains promote the non-canonical inflammasome via UBXN1

**DOI:** 10.21203/rs.3.rs-10666957/v1

**Published:** 2026-08-25

**Authors:** Duomeng Yang, Jason G. Cahoon, Tingting Geng, Chengliang Wang, Andrew G. Harrison, Evelyn Teran, Jack L. Wang, Yanlin Wang, Anthony T. Vella, Vijay A. Rathinam, Jianbin Ruan, Penghua Wang

**Affiliations:** 1 Department of Immunology, School of Medicine, UConn Health, Farmington, CT 06030, USA.; 2 Department of Medicine, School of Medicine, UConn Health, Farmington, CT 06030, USA.; 3 Current address: Department of Pathophysiology, Key Laboratory of State Administration of Traditional Chinese Medicine of the People’s Republic of China, School of Medicine, Jinan University, Guangzhou, Guangdong, China; 4 Current address: Harvard Medical School, Boston Children’s Hospital, Division of Immunology, Boston, MA, USA

**Keywords:** Inflammasome, UBXN1, Caspase-4, Ubiquitination, Unanchored ubiquitin chain

## Abstract

The non-canonical caspase-4 inflammasome is a crucial anti-bacterial immune mechanism, yet, when dysregulated, may contribute to sepsis pathogenesis. Its regulation is heavily reliant on transcriptional control of caspase-4 expression. However, posttranslational regulation of the caspase-4 inflammasome remains poorly understood. Here, we report that UBX domain-containing protein 1 (UBXN1) facilitates the non-canonical inflammasome via unanchored lysine 48- or 63-linked polyUb (K48/63-Ub) chains. UBXN1 deficiency impairs the LPS-induced caspase-4 inflammasome and pyroptosis, renders mice resistant to LPS and polymicrobial sepsis. Depleting cellular unanchored polyUb with ubiquitin-specific proteinase 5 (USP5) reduces, while inhibiting USP5 enhances, caspase-4 activation in a UBXN1-dependent manner. *In vitro*, unanchored K48/63-Ub chains enhance LPS-induced caspase-4 enzymatic activity in a chain length- and UBXN1-dependent manner. Mechanistically, UBXN1 directly interfaces with and bridges unanchored K48/63-Ub and caspase-4, forming a tripartite complex to facilitate caspase-4 activation. Our findings uncover a previously unrecognized UBXN1- and unanchored K48/63-Ub-dependent regulatory layer in the caspase-4 inflammasome.

## INTRODUCTION

Inflammasomes are large cytosolic multiprotein complexes formed in response to infections and cellular stresses, leading to auto-activation of inflammatory caspases (human caspase-1, −4, −5, murine caspase-1, −11), secretion of inflammatory mediators [Interleukin (IL) −1 and −18)] and pyroptosis, a form of cell death ^1^. Canonical inflammasomes are assembled by one of the NLRs [Nucleotide-binding and oligomerization domain (NOD), leucine-rich repeat (LRR)-containing receptors (NLRs)], AIM2 (absent in melanoma 2) or Pyrin, upon binding by a microbial ligand or cellular danger signal. They then oligomerize, recruit the adaptor ASC (Apoptosis-associated speck-like protein containing a caspase recruitment domain) (optional), then caspase-1, which undergoes self-cleavage into an active form. Active caspase-1 then proteolytically converts pro-IL-1β and pro-IL-18 into their biologically active, secreted forms ^2^. The non-canonical inflammasomes are activated by direct binding of a bacterial endotoxin, lipopolysaccharide (LPS), to human caspase-4/5, which preferably cleaves pro-IL-18, whereas murine caspase-11 can’t. Regardless of their preference for different cytokines, all the inflammatory caspases cleave Gasdermin D (GSDMD) into an active N-terminal fragment that forms pores in the plasma membrane, leading to cell death ^3^.

Although inflammasomes are critical for the control of many microbial infections, hyperactive inflammasome signaling may lead to sepsis as well as autoinflammatory / autoimmune conditions ^4^. For example, the caspase-11 inflammasome is the main driver of LPS and polymicrobial sepsis in mice ^5-8^, and caspase-4 inflammasome-mediated pyroptosis facilitates immunocoagulation and organ damage in human sepsis ^9-11^. Therefore, inflammasome signaling is subjected to tight regulation on both the transcriptional and posttranslational levels. In particular, ubiquitination is one of the most important posttranslational regulations for numerous immune signaling pathways, including the canonical NLRP3 inflammasome ^12^. Many ubiquitin E3 ligases have been identified to either inhibit NLRP3 signaling via degradation ^13-17^ or promote NLRP3-caspase-1 activation via lysin (K) 63-linked polyubiquitination ^18-20^. However, little is known about the function of ubiquitination in the non-canonical inflammasomes ^21^. Only one E3 ligase, neural precursor cell expressed developmentally down-regulated protein 4 (NEDD4), was reported to induce K48-linked polyubiquitination and subsequent degradation of caspase-11 ^22^. Whether ubiquitination is required for the activation of caspase-4 and −11 inflammasomes remains unknown.

By immunoprecipitation coupled with mass spectrometry, we identified ubiquitin regulatory x domain-containing protein 1 (UBXN1) and ubiquitin as caspase-4 interacting proteins. UBXN1 deficiency leads to impaired activation of caspase-4/11 and GSDMD, secretion of inflammasome-dependent cytokines and pyroptosis in response to intracellular LPS in HeLa cells and primary mouse macrophages. However, UBXN1 is dispensable for the NLRP3 inflammasome. UBXN1-deficient mice are protected from LPS- and cecal-ligation-and-puncture (CLP)-induced sepsis, evidenced by reduced mortality and systemic inflammation, compared to UBXN1-sufficient littermates. Mechanistically, UBXN1 interfaces with and bridges unanchored K48/63-linked ubiquitin chains (K48/63-Ub) and caspase-4, forming a complex to promote caspase-4 activation. In a cell-free system, depleting cellular unanchored polyubiquitin chains with recombinant ubiquitin-specific proteinase 5 (USP5) reduces, while inhibiting USP5 enhances, the LPS-induced caspase-4 inflammasome in a UBXN1-dependent manner. *In vitro*, unanchored K48/63-Ub chains enhance LPS-induced caspase-4 enzymatic activity in a chain length- and UBXN1-dependent manner, while neither mono-Ub nor K11-Ub can.

## RESULTS

### UBXN1 interacts with caspase-4/11

**Fig.1.**
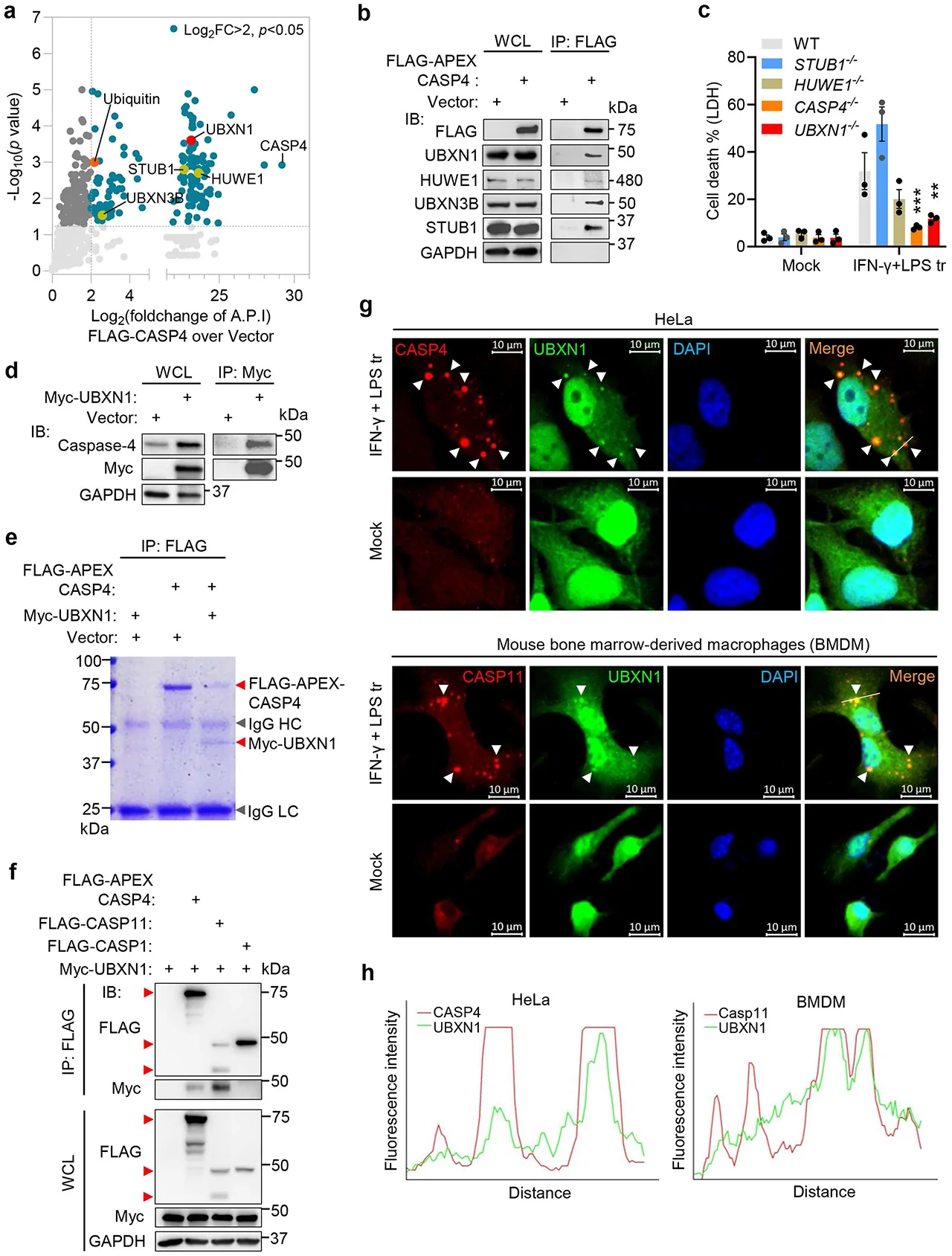
UBXN1 interacts with caspase-4/11. (**a**) A scatter plot showing the endogenous proteins co-immunoprecipitated (IP) with FLAG-CASP4 and identified by mass spectrometry. Proteins involved in ubiquitination are labeled. Dark green dots indicate the proteins with the Log_2_ foldchange (FC) of Average Precursor Intensity (API) > 2, *p* <0.05 by two-tailed Student’s *t*-test with Benjamini–Hochberg. The Log_2_FC is calculated as Log_2_ (API of a protein from the FLAG-CASP4 / Vector group). (**b**) Co-IP of FLAG-APEX-caspase-4 and endogenous interactors with an anti-FLAG antibody from HeLa cells transfected with a FLAG-APEX-CASP4 plasmid. Shown are the immunoblots (IB) of indicated proteins. WCL, whole cell lysate. (**c**) The percent cell death (LDH release) of HeLa cells primed with 10 ng/mL of human IFN-γ for 12 h and trasnfecetd with 1 μg/mL LPS (LPS tr) for 5 h. Mock are untreated controls. Bar, mean ± S.E.M, where each symbol represents a biological replicate, **p<0.01, ***p<0.001 by two-way ANOVA with the Dunnet comparison test. (**d**) Co-IP of Myc-UBXN1 and endogenous caspase-4 with an anti-Myc antibody from HeLa cells transfected with a Myc-UBXN1 plasmid. (**e**) Co-IP of FLAG-APEX-caspase-4 and Myc-UBXN1 with an anti-FLAG antibody from HeLa cells transfected with the indicated plasmids. Shown is Coomassie blue staining. IgG HC, immunoglobin heavy chain; LC, light chain. (**f**) Co-IP of FLAG-caspase and Myc-UBXN1 with an anti-FLAG antibody from HEK293T cells transfected with the indicated plasmids. The red arrow heads indicate desired protein bands. (**g**) Immunofluorescent staining of the indicated proteins in HeLa cells and mouse bone marrow-derived macrophages (BMDM) that were primed with IFN-γ and transfected with LPS. Mock, transfection reagent only. DAPI stains DNA. The arrows point to colocalizations. Scale bar: 10 μm. The lines are used for calculating spatial distribution of fluorescence intensity in (**h**). (**h**) Histogram analyses of the fluorescence intensity distribution in (**g**). The fluorescence intensity and distance were acquired by the white lines crossing the bright puncta in (**g**) using ImageJ. The results are representative of 2–3 indepedent experiemnts.

Ubiquitination is a major posttranslational regulation for immune signaling, however, little is known about its function in the non-canonical inflammasomes ^3^. To identify ubiquitination-related interactors of caspase-4 in an unbiased manner, we expressed FLAG-CASP4 in HeLa cells (a cancerous human epithelial cell line suitable for studying the non-canonical inflammasome), performed immunoprecipitation (IP) with an anti-FLAG antibody, and identified caspase-4-bound endogenous proteins by mass spectrometry. We identified 161 putative caspase-4 binders, however, only 5 are involved in ubiquitination, including two UBXN family members (UBXN1 and UBXN3B), two E3 ligases [STUB1 (STIP1 homology and U-box containing protein 1) and HUWE1 (HECT, UBA and WWE domain containing 1)], and ubiquitin (RPS27A) (Fig.1a, b). To examine their role in caspase-4 activation, we generated stable polyclonal *UBXN1*, *STUB1*, *HUWE1*, and *CASP4* knockout HeLa cells by CRISPR-Cas9 (Supplemental **Fig.S1a**). The UBXN3B phenotype was investigated with bone marrow-derived macrophages (BMDMs) derived from our previously generated tamoxifen-inducible knockout mouse model ^23-25^. We noted an obvious difference in cell death between *UBXN1*^−/−^ and its corresponding WT cells following LPS transfection, like *CASP4*^−/−^ (Fig. 1c, Supplemental **Fig.S1b**). Thus, we sought to focus on UBXN1 function in the caspase-4 inflammasome. Next, we validated the caspase-4-UBXN1 interaction by co-immunoprecipitating endogenous caspase-4 and Myc-UBXN1 with an anti-Myc antibody (Fig.1d). To estimate the caspase-4-UBXN1 binding capacity, we overexpressed FLAG-APEX-CASP4 and Myc-UBXN1 together and immunoprecipitated FLAG-APEX-CASP4. We observed that nearly an equal amount of Myc-UBXN1 was co-immunoprecipitated (Fig.1e). We confirmed that caspase-11, the murine counterpart of human caspase-4, was also able to pull down UBXN1, but human caspase-1, caspase-2 and caspase-7 could not (Fig.1f, Supplemental **Fig.S1c**), indicating that UBXN1 specifically targets the non-canonical inflammasome. Next, to validate the above immunoprecipitation results, we investigated endogenous UBXN1-caspase-4/11 colocalization by confocal microscopy. In resting cells (mock), caspase-4/11 was largely diffused throughout the cytoplasm, while UBXN1 was dispersed in the cytoplasm and nucleoplasm. Notably, following LPS transfection, UBXN1 was colocalized with caspase-4/11 to large punctate structures, typical of inflammasomes, in HeLa and primary murine macrophages respectively (Fig.1g, h, Supplemental **Fig.S2a**). These data suggest that UBXN1 is a component of the caspase-4/11 inflammasome complex.

### UBXN1 is crucial for the non-canonical inflammasome signaling

**Fig. 2.**
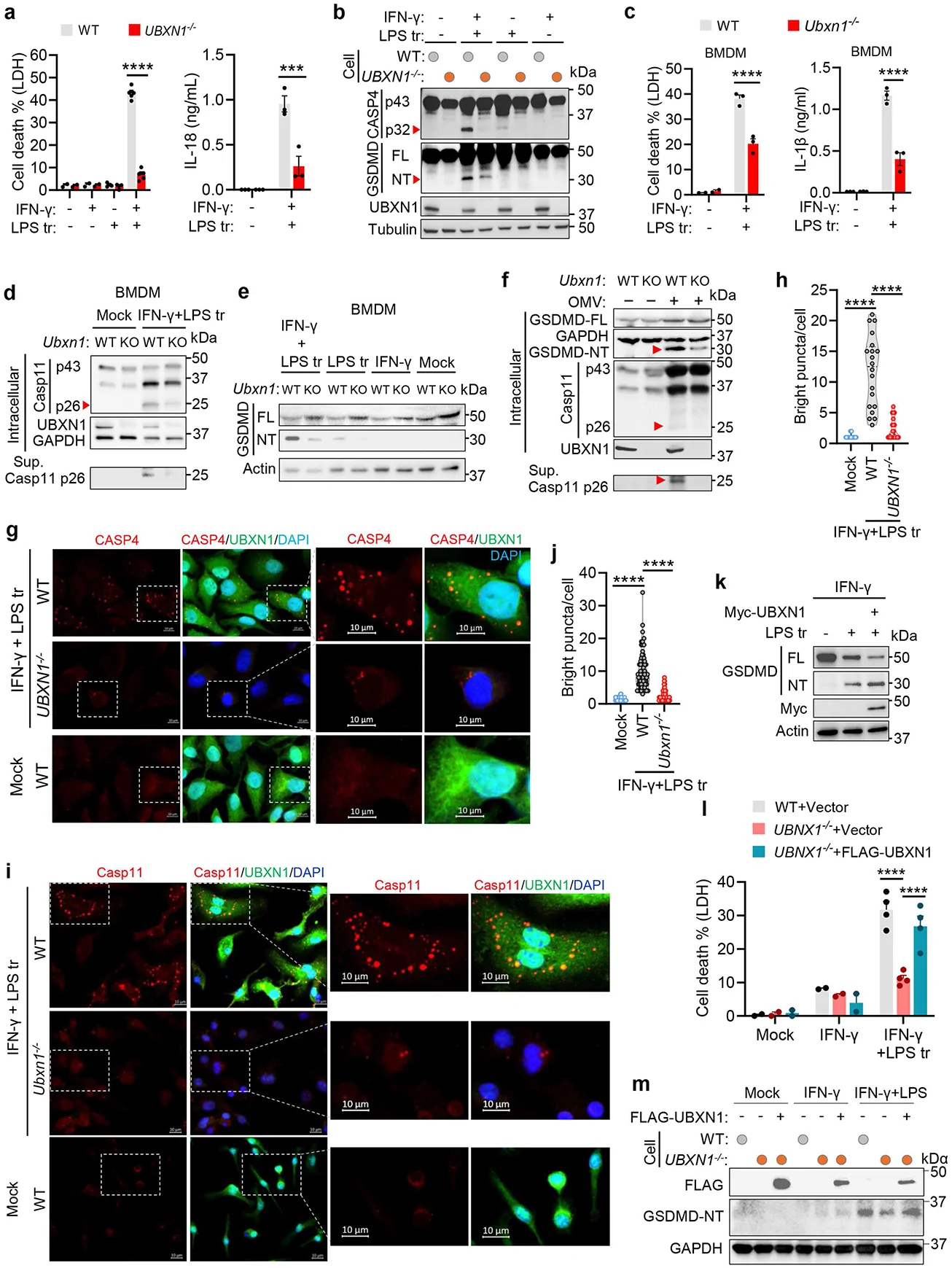
UBXN1 is critical for non-canonical inflammasome signaling. (**a**) The percent cell death and concentrations of IL-18 in the culture medium of HeLa cells that were primed with (+) / without (−) 10 ng/mL of human IFN-γ for 12 h and/or transfected with 1 μg/mL LPS (LPS tr) for 5 h. Each symbol represents a biological replicate, N=5 for IFN-γ+LPS in the cell death assay, N=3 for IL-18 ELISA assay. (**b**) The immunoblots of caspase-4 and GSDMD in HeLa cells treated as in (**a**). The red arrow heads indicate the cleaved products. FL, full-length; NT, N-terminal fragment. (**c**) The percent cell death of and IL-1β release from bone marrow-derived macrophages (BMDMs) that were primed with murine IFN-γ (10 ng/mL) for 3 h and then transfected with LPS (1 μg/mL) (LPS tr) for 16 h. N=3 mice/group. (**d**, **e**) The immunoblots of caspase-11 and GSDMD in BMDMs treated as in (**c**). Sup: the supernatant of cell culture. (**f**) The immunoblots of caspase-11 and GSDMD in BMDMs that were treated with outer membrane vesicles (OMV) derived from *E. coli* for 12 h. The red arrow heads indicate the cleaved fragments. (**g**) Immunofluorescent staining of the indicated proteins in HeLa cells treated as in (**a**). A region of interest (ROI) from each image is enlarged. Mock: transfection reagent only. Scale bar: 10 μm. (**h**) The violin plot of bright caspase-4 puncta per cell from (**g**). N=20 cells from 5 radom microscopic fields of view per group. (**i**) Immunofluorescent staining of the indicated proteins in BMDMs treated as in (**d**). A ROI from each image is enlarged. Scale bar: 10 μm. (**j**) The violin plot of bright caspase-11 puncta per cell from (**i**). N=70 cells from 5 radom microscopic fields of view per group. (**k**) The immunoblots of GSDMD in HeLa cells that were transfected with Myc-UBXN1 for 24 h, then treated with human IFN-γ and/or LPS as in (**a**). (**l**) The percent death of HeLa cells that were transfected with a FLAG-UBXN1 or vector plasmid, primed with IFN-γ and transfected with LPS. (**m**) The immunoblots of indicated proteins in HeLa cells from (**l**). Bar, mean ± S.E.M, ***p<0.001, ****p<0.0001 by two-tailed unpaired Student’s *t*-test (**a**, **c**) or one-way ANOVA with Tukey’s multiple comparisons test (**h**, **j, l**). The results are representative of 2-3 indepedent experiemnts.

Next, we in-depth characterized the role of UBXN1 in the non-canonical inflammasome signaling. We generated multiple monoclonal knockout lines and recapitulated the reduction of pyroptosis (Supplemental **Fig.S1d-f**, Fig.2a). Consistent with the cell death result, both active caspase-4 p32 and GSDMD-N fragments appeared in WT cells after LPS transfection, while their levels were greatly reduced in *UBXN1*^−/−^ cells (Fig.2b) as were maturation and secretion of IL-18 (Fig.2a). Moreover, UBXN1 expression was induced by IFN-γ and IFN-β (Supplemental **Fig.S1g**).

Next, we wanted to recapitulate the results from HeLa cells using primary BMDMs. Because of potential embryonic lethality of the *Ubxn1* null mutation ^26^, a tamoxifen-inducible global knockout mouse model was generated by crossing *Ubxn1*^*flox*/flox^ (flanking exons 3-9) with a Cre recombinase-estrogen receptor T2 (ERT2) line (Supplemental **Fig.S2b**, **c**). To minimize heterogeneity among individual mice, we pooled BMDMs from three ERT2-Cre^+^ *Ubxn1*^*flox*/flox^ mice, treated half of the cells with 4-hydroxyl (OH) tamoxifen to induce *Ubxn1* gene deletion (*Ubxn1*^−/−^) and the other half with the solvent (dimethyl sulfoxide, DMSO) (WT) *in vitro* (Supplemental **Fig.S2d**). Indeed, cell death, IL-1β secretion, activation of caspase-11 (both intracellular and secreted p26) and GSDMD (N-terminal fragment) were reduced in *Ubxn1*^−/−^ compared to WT BMDMs following LPS transfection (Fig.2c-e). The same result was observed when the caspase-11 inflammasome was activated by *E. coli*-derived outer membrane vesicles (OMVs) (Fig.2f). Moreover, by confocal microscopy we observed far fewer caspase-4/11 puncta in UBXN1 knockout than WT HeLa cells and BMDMs, respectively (Fig.2g-j; Supplemental **Fig.S2e**). Contrasting to UBXN1 knockout, overexpression of UBXN1 in WT cells enhanced GSDMD cleavage following LPS transfection (Fig.2k). Trans-complementation of UBXN1 restored LPS-elicited cell death and GSDMD cleavage to *UBXN1*^−/−^ cells (Fig.2l, m). In summary, these results show that UBXN1 positively regulates the non-canonical inflammasome in human and murine cells.

### UBXN1 is dispensable for IFN-γ, TLR4 and NLPR3 inflammasome signaling

To trigger robust non-canonical inflammasome signaling, BMDMs and HeLa cells were primed with IFN-γ followed by transfecting LPS. IFN-γ signaling induces GSDMD, caspase-4/11 and guanylate binding protein (GBPs) etc., while LPS stimulates TLR4 signaling that can also prime inflammasomes. Therefore, we asked if UBXN1 targets the IFN-γ receptor-JAK1 (Janus kinase 1)-STAT1 (signal transducer and activator of transcription) or TLR4 signaling pathways. We observed no significant differences in JAK1 and STAT1 phosphorylation, *Tnf* or *Casp11* mRNA levels in IFNγ-primed WT and *Ubxn1*^−/−^ BMDMs (Supplemental **Fig.S3a**, **b**). Moreover, human IFNγ-induced GBP expression was comparable between WT and *UBXN1*^−/−^ HeLa cells (Supplemental **Fig.S3c**, **d**). These observations demonstrate that UBXN1 is not involved in IFN-γ signaling. LPS-induced *Tnf* mRNA expression was slightly enhanced in *Ubxn1*^−/−^ BMDMs (Supplemental **Fig.S3e**). Overall, these results suggest that UBXN1 directly regulates the non-canonical inflammasome pathway and not through the feed-forward TLR4 signaling pathway.

Next, we examined if UBXN1 plays a role in canonical inflammasomes. We induced the NLRP3 inflammasome with LPS+ATP or nigericin, NLRC4 inflammasome with LPS+flagellin in primary BMDMs. Secretion of IL-1β and IL-18, caspase-1 activation (p10), and cell death were similar between WT and *Ubxn1*^−/−^ cells (Supplemental **Fig.S3f-h**), and LPS-induced NLRP3 expression was also similar (Supplemental **Fig.S3i**). Hence, UBXN1 is dispensable for the NLRP3/NLRC4 inflammasome signaling.

### UBXN1 facilitates the pathogenesis of LPS and polymicrobial sepsis in mice

**Fig. 3.**
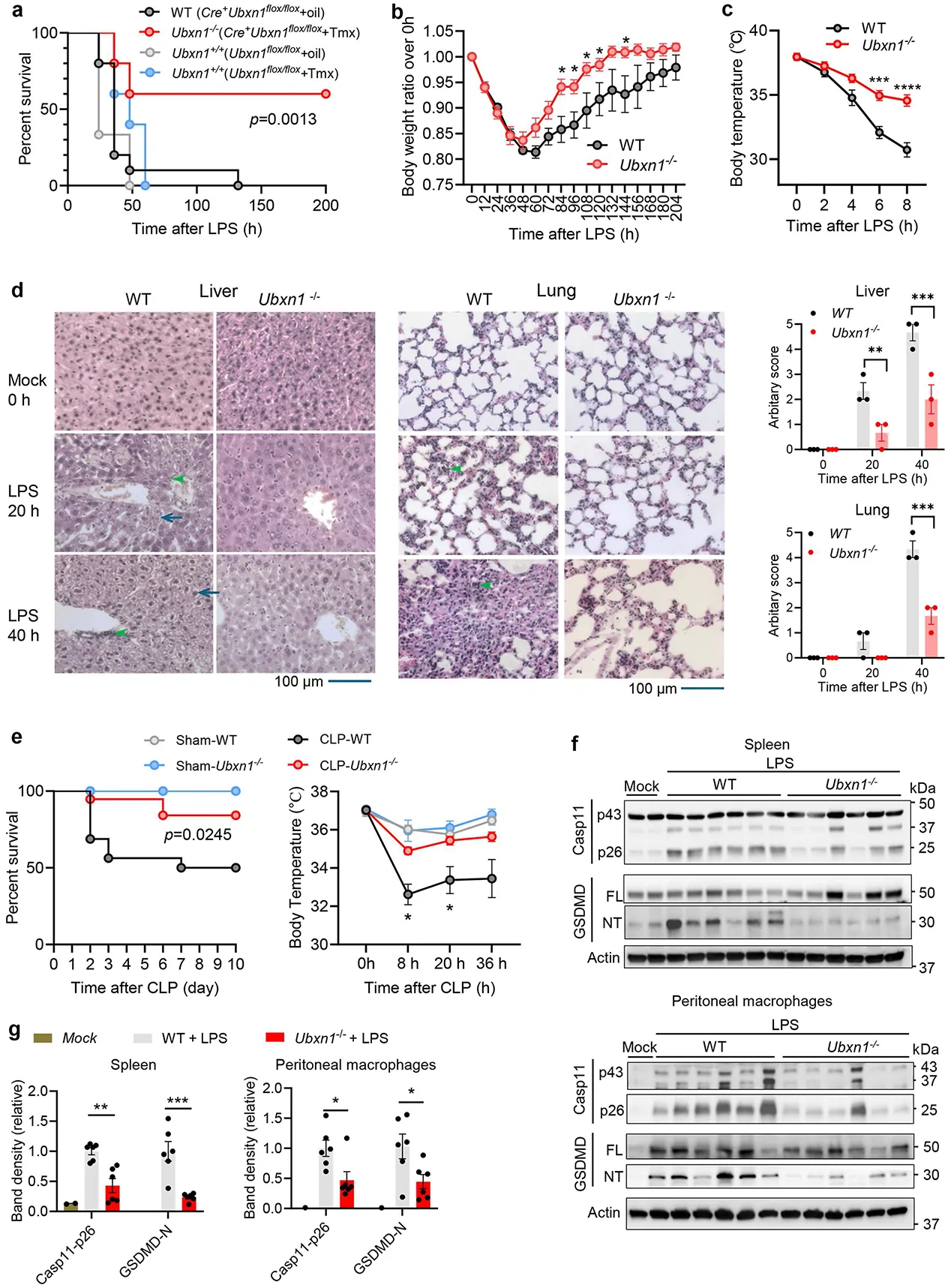
*Ubxn1*^−/−^ mice are resistant to lethal LPS and CLP sepsis (**a**) The survival curves of age- and sex-matched littermates injected with LPS (12 mg/kg) intraperitoneally. N=6 mice for *Ubxn1*^*flox/flox*^ +oil, 5 for *Ubxn1*^*flox/flox*^ +tamoxifen (Tmx), 10 for WT (Cre^+^*Ubxn1*^+/+^+oil) and *Ubxn1*^−/−^ (Cre^+^*Ubxn1*^+/+^+Tmx) respectively. *p*=0.0013 (Log-Rank test, WT vs. *Ubxn1*^−/−^). (**b**) The body mass change of WT and *Ubxn1*^−/−^ littermates treated with 8 mg/kg of LPS. N=10 mice/genotype. (**c**) The rectal temperatures of WT and *Ubxn1*^−/−^ littermates treated with 10 mg/kg of LPS. N=8 mice per group. (**d**) The hematoxylin and eosin staining (H&E) from mice administered 10 mg/kg of LPS. The microscopic fields are taken from larger tissue sections in Supplemental Fig.S7c. The arbitrary disease scores range from 1 to 5 with 5 representing the worst damage / immune infiltration. N=3/group. The arrow heads indicate immune infiltrates; arrows point to hepatocytic vacuolation. (**e**) The survival curves and rectal temperatures of age- and sex-matched littermates subjected to cecal ligation and puncture (CLP) sepsis. In the survival curves, N=16 mice for WT and 19 for *Ubxn1*^−/−^, *p*=0.0245 (Log-Rank test). In the temperature curves, N=4 mice for Sham/group and 6 for CLP/group. (**f**) The immunoblots of caspase-11 and GSDMD in age- and sex-matched littermates that were treated with 10 mg/kg of LPS intraperitoneally for 36 h. Each lane represents one mouse. Mock, mice treated with phosphate-buffered saline; FL, full-length; NT, N-terminal fragment. (**g**) Semi-quantification of the protein band density in (**f**). The Y-axis shows the ratios of p26 or GSDMD-NT bands (normalized with actin) of each group relative to those of the WT+LPS group. N=6 mice for the LPS group and 2 for Mock. Bar, mean ± S.E.M, *p<0.05, ***p<0.001, ****p<0.0001 by unpaired Student’s *t*-test for (**b**, **c, g**) and the right panel of (**e**), or two-way ANOVA with Tukey’s multiple comparisons for (**d**). The results are representative of 2-3 indepedent experiemnts.

The aforementioned *in cellulo* results clearly demonstrate an important role for UBXN1 in the non-canonical inflammasome, but its role *in vivo* is unknown and thus we characterized UBXN1 function during caspase-11-mediated sepsis ^5-7,27^. After five doses of tamoxifen (Tmx) in ERT2-Cre^+^*Ubxn1*^flox/flox^ mice, UBXN1 protein was effectively depleted in most tissues except the brain (Supplemental **Fig.S4a**). This is due to inefficient penetration of Tmx across the blood brain barrier. Nonetheless, this would not impact our study on sepsis. *Ubxn1*^−/−^ (ERT2-Cre^+^*Ubxn1*^flox/flox^ + Tmx) mice showed no discernible, developmental, or behavioral abnormalities, when compared to WT or *Ubxn1*^+/+^ (ERT2-Cre^+^*Ubxn1*^flox/flox^ + oil, *Ubxn1*^flox/flox^ + Tmx, *Ubxn1*^flox/flox^ + oil). The major blood immune populations were the same between *Ubxn1*^−/−^ and WT mice (Supplemental **Fig.S4b, c**). Importantly, *Ubxn1*^−/−^ animals, like *Casp11*^−/−^ were better protected from lethal LPS sepsis than WT or *Ubxn1*^+/+^ littermates (Fig.3a, Supplemental **Fig.S5a**). There was no difference in mortality between *Ubxn1^flox/flox^* +Tmx and *Ubxn1^flox/flox^* + oil mice (Fig.3a), excluding any side effect of tamoxifen (which was cleared before LPS challenge) on LPS sepsis. Therefore, we used only one WT littermate control, *i.e*., ERT2-Cre^+^*Ubxn1*^flox/flox^ + oil in subsequent experiments. Both WT and *Ubxn1*^−/−^ mice lost ~18% of body weight by 48 h after LPS, however, the knockout animals recovered faster than WT mice (Fig.3b). The body temperature of *Ubxn1*^−/−^ mice dropped more slowly than that of WT mice after LPS (Fig.3c). At 36 h after LPS, WT mice tended to huddle together at cage corners, while the knockout animals were more active and agile (Supplemental **Fig.S5b** and **video**). Consistent with lethal sepsis, significant tissue damage was also noted in the lung and liver of WT mice. Thickened alveolar septa, reduced alveolar spaces, massive cellular infiltration in alveolar septa and collapsed alveoli, hepatocytic vacuolation, and diffusing hemorrhage were evident in WT mice at 20 and 40 h after LPS, compared to 0 h. However, the damage to the lung and liver in *Ubxn1*^−/−^ mice was much less severe than WT (Fig.3d, Supplemental **Fig.S5c**). In addition to the LPS endotoxemia model, the cecal ligation-and-puncture (CLP) induces polymicrobial, partially inflammasome-dependent lethal sepsis in mice ^8^ that most closely represents the progression and characteristics of human sepsis ^28^. Consistently, the *Ubxn1*^−/−^ animals had better survival after induction of CLP sepsis than did the WT littermates. The body temperatures of *Ubxn1*^−/−^ mice with CLP were also higher than those of WT mice with CLP. As negative controls, all the sham mice survived (Fig.3e). These results demonstrate that UBXN1 deficiency protects mice from sepsis.

**Fig. 4.**
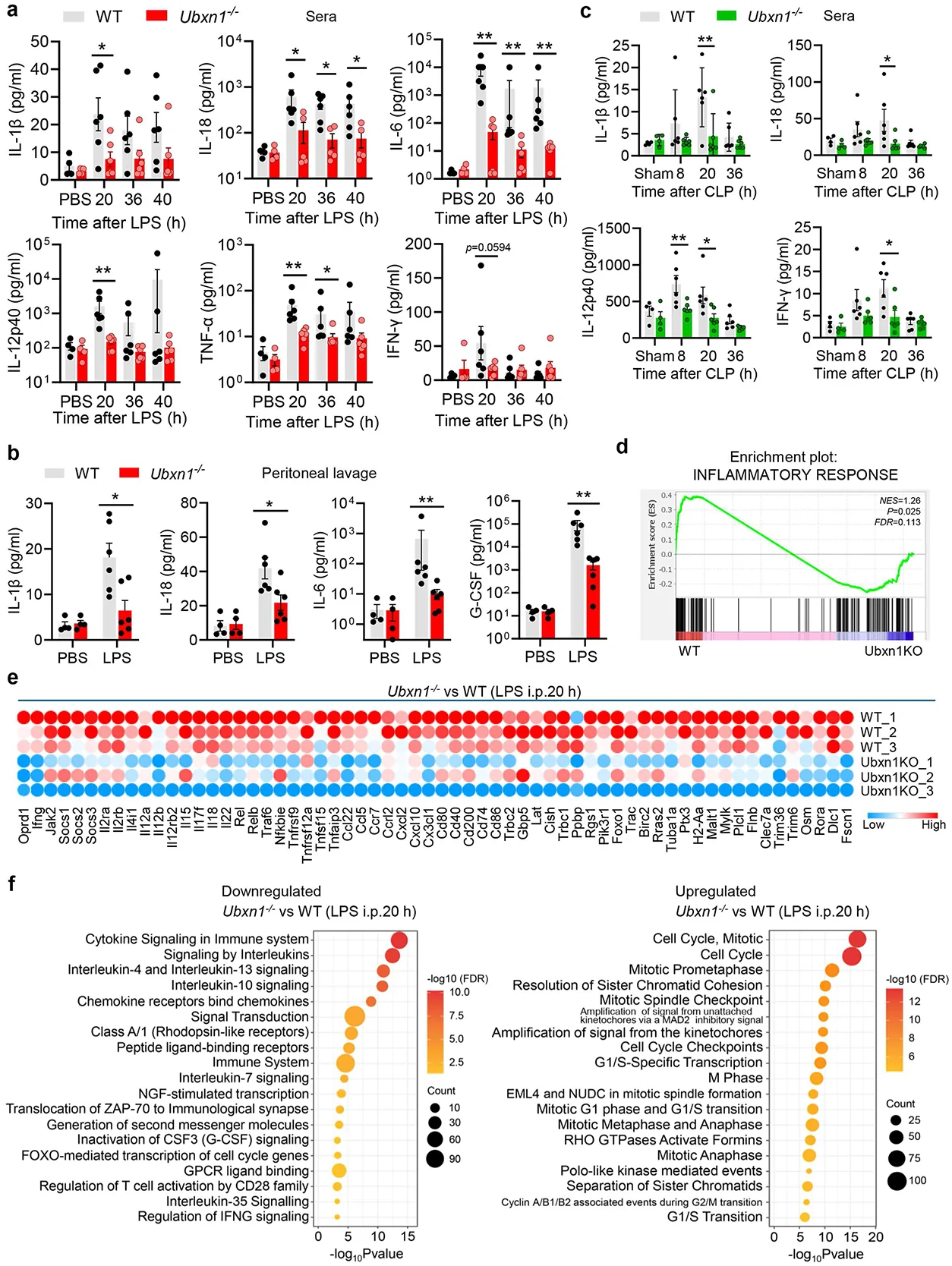
*Ubxn1*^−/−^ mice present reduced inflammatory responses during sepsis. (**a**, **b**) The concentrations of cytokines/growth factors in the (**a**) sera at various timepoints and (**b**) peritoneal lavages at 40 h in age- and sex-matched littermates who received 10 mg/kg of LPS or phosphate-buffered saline (PBS) intraperitoneally. (**c**) The concentrations of serum cytokines in age- and sex-matched littermates subjected to sham or a cecal ligation-and-puncture (CLP) procedure. (**d-f**) The whole transcriptome analysis by RNA-sequencing. Age- and sex-matched WT and *Ubxn1*^−/−^mice were treated with 10 mg/kg of LPS intraperitoneally. At 0, 8 and 20 h after LPS, splenic macrophages were purified and subjected to RNA-sequencing. N=3 mice per group. Differentially expressed genes (DEGs) were determined by two-tailed unpaired Student’s t-test, Log_2_foldchange≧1 (upregulated) or ≦−1 (downregulated), *p. adjust*<0.05. (**d**) The gene set enrichment analysis (GSEA) of a significant gene set of inflammatory response. The analysis was performed using GSEA version 2.0.9. FDR, false discovery rate; NES, normalized enrichment score. (**e**) A panel of genes associated with inflammatory responses. The colors indicate expression level. (**f**) The top Reactome pathways enriched from differentially expressed genes (Log_2_fold change ≥1 or ≤ −1, *p.adjust*< 0.05). FDR, false discovery rate; Log_10_Pvalue by right-tailed Fisher’s exact *t* test with Benjamini & Hochberg. Bar, mean ± S.E.M, where each dot represents one animal, *p<0.05, **p<0.01 by two way-ANOVA with the Dunnett comparison.

Considering the crucial role of the non-canonical inflammasome in both LPS and CLP sepsis pathogenesis ^5-8^, we investigated caspase-11 and GSDMD activation in mice. Indeed, the levels of active p26 fragment of caspase-11 and N-terminal fragment of GSDMD were lower in the spleen and peritoneal macrophages of *Ubxn1*^−/−^ mice than those in WT following LPS administration (Fig.3f, g). Consequently, the concentrations of inflammasome-dependent IL-1β and IL-18 were reduced in the sera and peritoneal lavages of *Ubxn1*^−/−^ mice at various timepoints after LPS (Fig.4a, b; Supplemental **Fig.S5d**). The secondary inflammatory mediators that can be induced by IL-1β and IL-18 were also reduced, including IL-6, IL-12, G-CSF, TNF-α etc. ^29-31^. However, IFN-β induction was comparable between WT and knockout mice, further suggesting the dispensable role of UBXN1 in TLR4 signaling. The same results were noted in the animals with CLP sepsis (Fig.4c; Supplemental **Fig.S5e**).

To gain a holistic picture of UBXN1 functions during LPS sepsis, we performed an RNA-sequencing analysis of macrophages (a major cell type of inflammasome signaling) of WT and *Ubxn1*^−/−^ spleens at various stages of LPS sepsis (0, 8 and 20 h). We observed fewer than 10 differentially expressed genes (DEGs) (*Ubxn1*^−/−^ vs WT), including down- and up-regulated genes (Log_2_ fold change ≧ 1 or ≦ −1, *p.adjust*<0.05), at 0 and 8 h after LPS, respectively. Intriguingly, at 20 h after LPS, 337 and 649 genes were down- and up- regulated in the knockout mice, respectively (Supplemental **Fig.S6a**). The down-DEGs were enriched in inflammatory responses, cytokines/chemokines and signaling (Fig.4d-f). Notably, consistent with the ELISA data (Fig.4a), *Ifng* (Log_2_= −4.1), *Il12b* (p40) (Log_2_= −3.0), *Il12a* (p35) (Log_2_ = −1.4) (p35), and *Il18* (Log_2_ = −1.1) were significantly downregulated (Fig.4e). At the mRNA level, *Il6*, *Tnf*, and *Csf2/3* were not significantly down-regulated in the splenic macrophage of *Ubxn1*^−/−^ mice, contrasting to their serum concentrations which were significantly lower in *Ubxn1*^−/−^ mice (Fig.4a). This may not be surprising because these genes are abundantly expressed by many types of tissue cells, where UBXN1 may be crucial. The up-DEGs were predominantly enriched in the cell cycle pathway (Fig.4f), consistent with attenuated sepsis in *Ubxn1^−/−^* mice. Indeed, LPS sepsis was associated with highly elevated cytokine/chemokine signaling, while significantly repressed cell cycle in WT mice at 8 and 20 h after LPS, compared to 0 h (Supplemental **Fig.S6b, c**). In summary, these animal studies show a significant role for UBXN1 in sepsis pathogenesis.

### UBXN1 participates in the caspase-4/11 inflammasome assembly

**Fig. 5.**
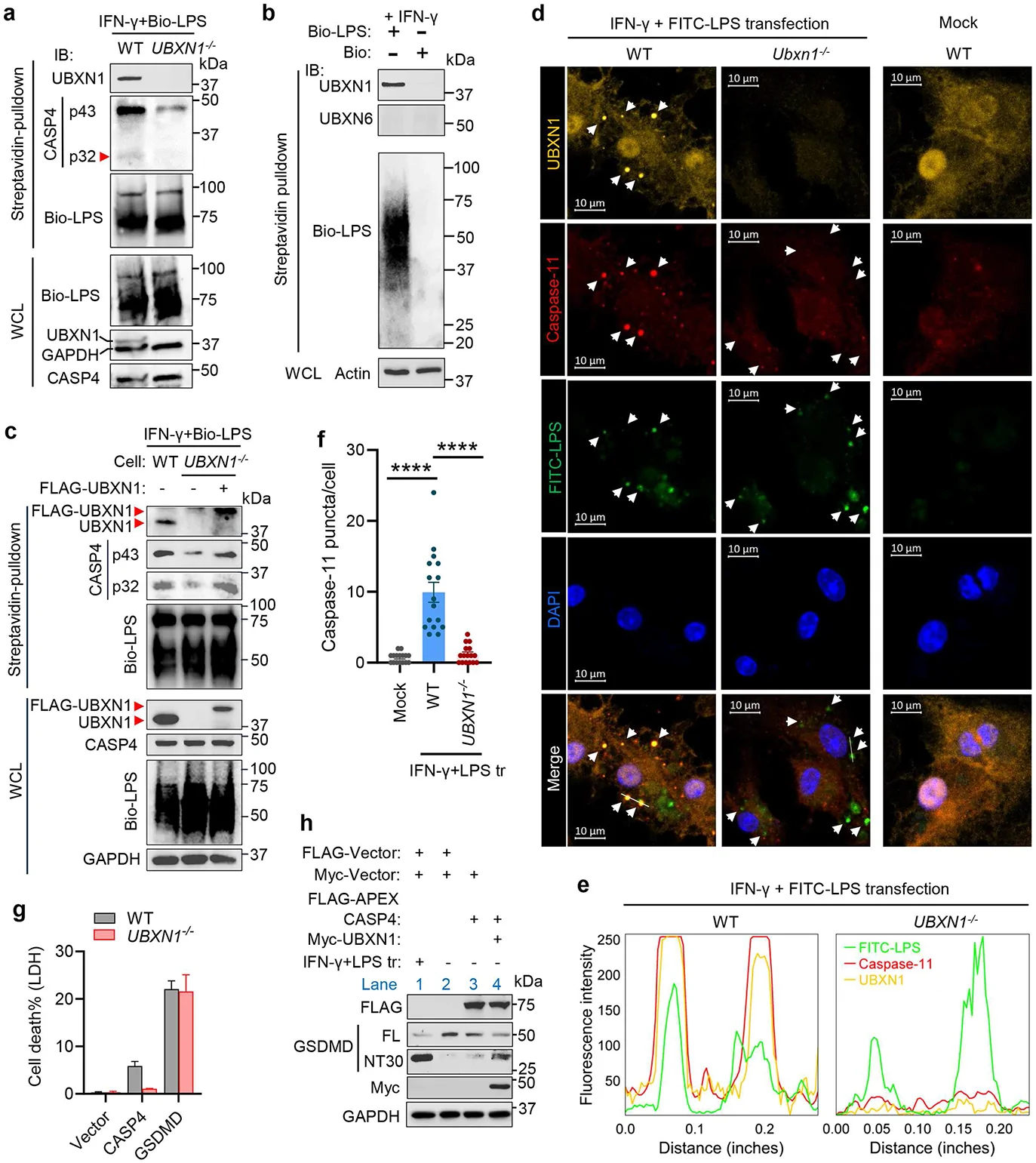
UBXN1 facilitates the caspase-4/11 inflammasome assembly (**a, b**) Streptavidin pulldown of biotinylated (Bio) LPS from HeLa cells that were primed with IFN-γ and transfected with biotin or Bio-LPS for 5 h. IB, immunoblot; WCL, whole cell lysate. (**c**) Streptavidin pulldown as in (**a**) except that cells were transfected with a FLAG-UBXN1 or vector plasmid before priming and LPS. (**d**) Immunofluorescent staining of the indicated proteins in BMDMs that were primed with IFN-γ and transfected with FITC-LPS. DAPI stains DNA. The arrows point to colocalizations of FITC-LPS, UBXN1 and caspase-11. Mock, transfection reagent only. Scale bar: 10 μm. The lines are used for measuring the colocalizations of LPS, caspase-11 and UBXN1 as in (**e**). (**e**) Histogram analyses of the fluorescence intensity distribution in (**d**) using ImageJ. The fluorescence intensity and distance were acquired by the white lines crossing the bright puncta in (**d**). (**f**) The numbers of bright caspase-11 puncta per cell from (**d**). Bar, mean ± S.E.M, N=16 cells/group, ****p<0.0001 by one-way ANOVA. (**g**) The percent death (LDH release) of HeLa cells transfected with a FLAG-CASP4 or - GSDMD plasmid for 24 h. (**h**) The immunoblots of indicated proteins in HeLa cells transfected with indicated plasmids for 24 h. IFN-γ priming plus LPS transfection (Lane 1) serves as the positive control for GSDMD cleavage. FL, full-length; NT, active N-terminal fragment. The results are representative of 2-3 indepedent experiemnts.

The aforementioned results suggest that UBXN1 directly regulates the caspase-4/11 inflammasome. Therefore, we reasoned that endogenous UBXN1 would be associated with the LPS-caspase-4 complex. To address this, we transfected HeLa cells with either biotin or biotin-conjugated LPS followed by streptavidin pulldown. Indeed, both UBXN1 and caspase-4 were pulled down together with biotin-LPS (Fig.5a, b). As a negative control, UBXN6, which shares similar functional domains with UBXN1, was not precipitated by biotin-LPS (Fig.5b). Of note, the amount of caspase-4 bound by biotin-LPS was significantly less in *UBXN1*^−/−^ than WT cells (Fig.5a). Trans-complementation of UBXN1 to *UBXN1*^−/−^ cells fully restored LPS-casapse-4 binding (Fig.5c). Consistently, in WT primary BMDMs, UBXN1 and caspase-11 co-localized extensively with FITC-LPS, while the caspase-11 puncta and caspase-11-LPS complexes were significantly reduced in *Ubxn1*^−/−^ cells (Fig.5d-f). These results suggest that UBXN1 participates in the assembly of the caspase-4/11 inflammasome complex. To address this finding more directly, we transiently overexpressed CASP4 or GSDMD in HeLa cells to induce pyroptosis without LPS. Caspase-4 overexpression alone in WT cells induced moderate death, which was reduced in *UBXN1*^−/−^ cells. However, GSDMD overexpression-induced cell death was not affected by *UBXN1* knockout, suggesting that UBXN1 directly targets caspase-4 (Fig.5g). In agreement with this, overexpression of Myc-UBXN1 enhanced FLAG-APEX-caspase-4-induced endogenous GSDMD cleavage (Lanes 4 vs 3) (Fig.5h).

### UBXN1 facilitates K48/63-Ub binding to caspase-4/11

**Fig. 6.**
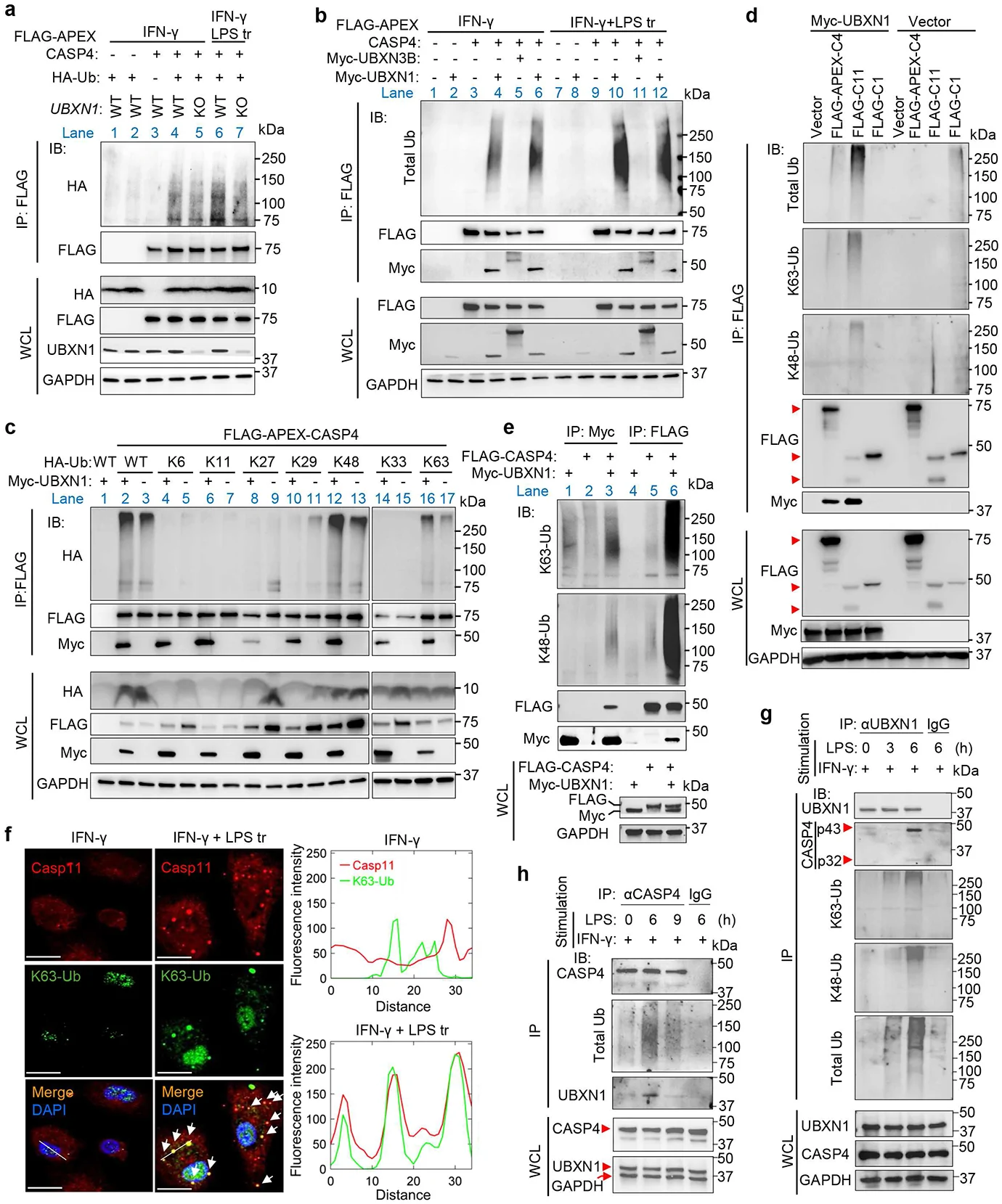
UBXN1 promotes K48/K63-Ub attachment to caspase-4/11. (**a**) Immunoprecipitation (IP) of FLAG-APEX-caspase-4 with an anti-FLAG antibody. HeLa cells were transfected with the indicated plasmids for 24 h, primed with IFN-γ for 12 h and/or transfected with LPS (LPS tr) for 5 h. (−) stands for an empty vector plasmid. Shown are the immunoblots (IB) of indicated proteins. WCL, whole cell lysate; KO, knockout. (**b**) IP of FLAG-APEX-caspase-4 with an anti-FLAG antibody from HeLa cells transfected with different combinations of indicated plasmids and corresponding vector (−) for 24 h, followed by treatment with human IFN-γ and LPS as indicated. The endogenous Ub was detected by a polyubiquitin antibody. (**c**) Co-immunoprecipitation (IP) of FLAG-APEX-caspase-4 with an anti-FLAG antibody from HeLa cells transfected with various combinations of FLAG-APEX-CASP4, Myc-UBXN1, HA-tagged WT or individual Kn-Ub plasmids. (−) stands for an empty vector plasmid. (**d**) IP of FLAG-caspase with an anti-FLAG antibody from HeLa cells that were transfected with the indicated plasmids. (**e**) IP of FLAG-caspase-4 and Myc-UBXN1 from HEK293T cells. Cells were transfected with the indicated plasmids for 24 h, then equally split for IP with an anti-Myc or anti-FLAG antibody separately. (**f**) Immunofluorescent staining of the indicated proteins in murine bone marrow derived macrophages that were primed with IFN-γ and transfected with LPS (LPS tr). The histograms show the colocalization between caspase-11 and K63-Ub. (**g**) IP of endogenous UBXN1 with an anti-UBXN1 antibody from HeLa cells that were primed with IFN-γ and transfected with LPS. IgG is a negative control. (**h**) IP of endogenous caspase-4 with an anti-caspase-4 antibody from HeLa treated as in (**g**). The results are representative of 2-3 indepedent experiemnts.

Several UBXNs including UBXN1 and UBXN3B are putative adaptor proteins that bridge ubiquitin ligases E_3_ and their substrates ^32^, interact with polyUb chains of a ubiquitinated (covalently modified) protein ^33^ or unanchored polyUb chains ^34^ through protein-protein interaction. Therefore, we asked if UBXN1 regulated caspase-4/11 function via ubiquitination. We first overexpressed FLAG-APEX-CASP4 and HA-Ub in HeLa cells and performed immunoprecipitation with an anti-FLAG antibody. Overexpression of HA-Ub enhanced polyUb modification to caspase-4 in both WT and *UBXN1*^−/−^ cells (Lanes 4 and 5 vs 3). Of note, LPS transfection further increased polyUb attachment to caspase-4 in WT (Lane 6), but not in *UBXN1*^−/−^ cells (Lane 7) (Fig.6a), suggesting that LPS-induced polyUb attachment to caspase-4 is dependent on UBXN1. Consistent with this, overexpression of UBXN1 increased polyUb attachment to caspase-4 even without LPS transfection (Lanes 4 and 6 vs 3); the ubiquitination was further enhanced by LPS (Lanes 10 and 12 vs 4 and 6). However, overexpression of UBXN3B did not (Lanes 5 and 11), demonstrating the specificity of UBXN1-mediated polyUb conjugation to caspase-4 (Fig.6b). To pinpoint the specific linkage of polyUb chains associated with caspase-4, we overexpressed HA-tagged WT or individual mutant K_n_-Ub, FLAG-APEX-CASP4, and Myc-UBXN1 (or vector) simultaneously. Each K_n_ mutant contains only one active K residue with the remaining K residues mutated to R (Supplemental **Fig.S7a**). UBXN1 overexpression enhanced only K48-Ub (Lanes 12 vs 13 in the IP panel), K63-Ub (Lanes 16 vs 17) as well as WT-Ub modifications to caspase-4 (Lanes 2 vs 3) when compared to the empty vector control (Fig.6c, Supplemental **Fig.S7b**). Next, we confirmed that endogenous K48/63-Ub, not the other linkages, were attached to caspase-4/11, but not caspase-1 (Fig.6d, Supplemental **Fig.S7c**).

The above results suggest that UBXN1 mediates K48/63-Ub modifications to caspase-4/11. We then asked if caspase-4 facilitated K48/63-Ub binding to UBXN1. We expressed Myc-UBXN1, FLAG-CASP4 individually or together, and performed immunoprecipitation with an anti-Myc or FLAG antibody. In Myc immunoprecipitation, FLAG-caspase-4 alone was a negative control. As shown in Fig.6e, the amount of endogenous K48/63-Ub associated with Myc-UBXN1 alone was marginally higher than FLAG-caspase-4 alone (Lanes 1 vs 2). Notably, K48/63-Ub association with Myc-UBXN1 was enhanced by the presence of FLAG-caspase-4 (Lane 3). Conversely, in FLAG immunoprecipitation, Myc-UBXN1 alone served as a negative control, and again co-expression of Myc-UBXN1 significantly increased K48/63-Ub attachment to FLAG-caspase-4 (Lanes 6 vs 5) (Fig.6e).

Next, we investigated the interactions among endogenous UBXN1, polyUb and caspase-4 under physiological conditions. We first confirmed that K63-Ub was colocalized with caspase-11 in primary BMDMs after LPS transfection (Fig.6f). We then noted that caspase-4 and K48/63-Ub were co-immunoprecipitated with UBXN1 with an anti-UBXN1 IgG after LPS transfection, and the binding increased with the time of LPS treatment, with the peak at 6 h (Fig.6g). Similarly, K48/63-Ub chains and UBXN1 were pulled down by caspase-4 with an anti-caspase-4 IgG following LPS stimulation (Fig.6h).

### Unanchored K48/63-Ub chains promote caspase-4/11 activation in a UBXN1-dependent manner

**Fig. 7.**
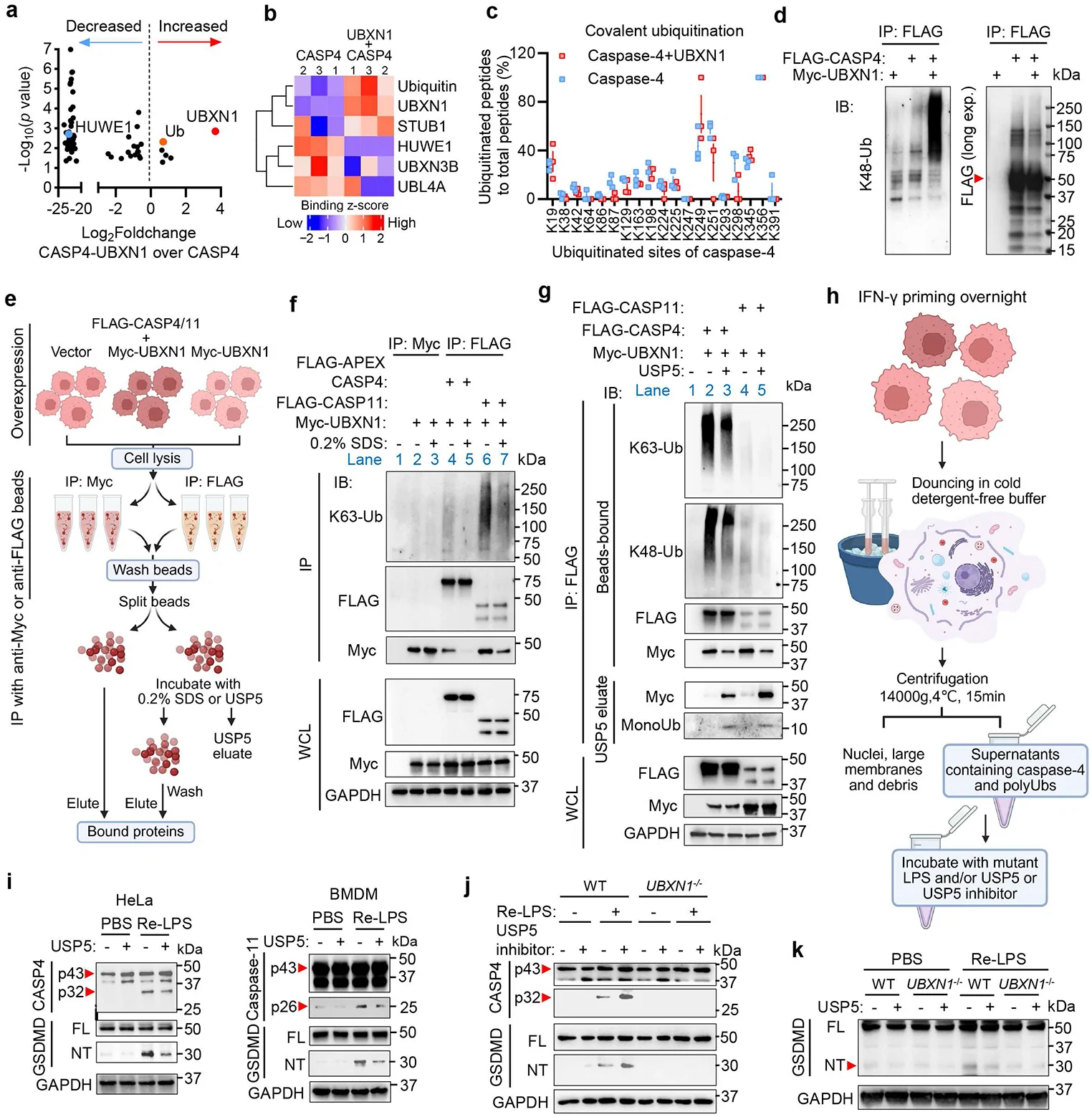
Unanchored K48/63-Ub chains bind to and activate caspase-4/11 in a UBXN1-dependent manner. (**a**) A scatter plot of FLAG-caspase-4 interactors with altered affinity in the presence of Myc-UBXN1. HeLa cells were primed with IFN-γ for 12 h, transfected with a FLAG-CASP4 and vector or Myc-UBXN1 plasmid for 24 h. Immunoprecipitation (IP) was performed with an anti-FLAG antibody and bound proteins were identified by mass spectrometry. Log_2_foldchange (FC) represents the ratio of average precursor intensity of a caspase-4 interactor in the FLAG-cAsP4 + Myc-UBXN1 to FLAG-CASP4 + vector group. Each dot represents a hit with *p*< 0.05 by two-tailed unpaired Student’s *t*-test with Benjamini–Hochberg, N=3 independent experiments. (**b**) The heatmap of binding Z-scores for ubiquitination-related proteins from (**a**). (**c**) The frequencies of covalent ubiquitination of individual caspase-4 lysine (K) residues from (**a**). The ubiquitination ratio of a K is expressed as percent (%) of modified peptide (di-glycine residue) over total peptide counts containing the corresponding K. Bar, mean ± S.E.M., N=3 independent experiments, *p*>0.05 by two-way ANOVA with the Bonferroni post-hoc test. (**d**) IP of FLAG-caspase-4 from HEK293T cells as in (**a**). IB, immunoblot. The arrowhead indicates unmodified FLAG-caspase-4. (**e**) The IP procedures to distinguish free polyubiquitin chain binding to caspase-4/11 from covalent ubiquitination of caspase-4/11. Created with BioRender.com. (**f, g**) The immunoblot of indicated proteins from (**e**), WCL, whole cell lysate. (**h**) The procedures to establish a cell-free system for studying the noncanonical inflammasome. Created with BioRender.com. (**i**) The immunoblots of indicated proteins in HeLa and bone marrow-derived macrophages (BMDMs) obtained from (**h**). Cells were primed with IFN-γ, douncing lysed, treated with Re-LPS/USP5 protein for 4 h. PBS, phosphate-buffered saline; FL, full-length; NT, N-terminal fragment. (**j**) The immunoblots of indicated proteins in HeLa cells treated as in (**i**) except that USP5 protein was replaced by an USP5 inhibitor. (**k**) The immunoblots of GSDMD in HeLa cell lysates from (**h**) incubated with a recombinant USP5 protein (50 ng/mL) and Re-LPS (500 μg/mL) or phosphate buffered saline (PBS) at 37°C for 4 h. The results are representative of 2-3 indepedent experiemnts.

Early in this study, we demonstrated that ubiquitin was co-immunoprecipitated with caspase-4 by unbiased co-IP and mass spectrometry, suggesting that polyubiquitin chains might interact with caspase-4 (Fig.1a). Traditionally, ubiquitination is regarded as the covalent modification of a protein by ubiquitin. However, cells also have unanchored polyUb chains that can noncovalently bind and regulate the function of target proteins ^35^. Therefore, we first asked if UBXN1 overexpression promoted covalent ubiquitination of caspase-4 and further attempted to identify its ubiquitination site by immunoprecipitation and mass spectrometry. Notably, overexpression of UBXN1 enhanced ubiquitin binding to caspase-4 (Fig.7a, b; Supplemental **Fig.S8a**, **b**). We observed nineteen ubiquitinated lysine residues in FLAG-caspase-4; however, none of them were significantly enhanced when Myc-UBXN1 was co-overexpressed (Fig.7c), suggesting that UBXN1 does not promote covalent conjugation of Ub to caspase-4. Consistent with this, when detected with an anti-FLAG antibody, the larger forms of FLAG-caspase-4 (including ubiquitinated forms) relative to FLAG-caspase-4 monomer was the same with/without Myc-UBXN1 (right panel, Fig.7d). However, we noted much more K48-Ub in the presence of Myc-UBXN1 (left panel, Fig.7d). These results strongly support the idea that UBXN1 chaperones free polyUb chains to caspase-4 via protein-protein interaction. To further confirm this, 0.2% sodium dodecyl-sulfate (SDS) was included in the immunoprecipitation assay to disrupt regular protein-protein interactions, but not the strong antibody-antigen interaction (Fig.7e). Indeed, 0.2% SDS did not affect immunoprecipitation of FLAG-caspase-4/11 with an anti-FLAG or Myc-UBXN1 with an anti-Myc antibody (IP, Lanes 2 vs 3, 4 vs 5, 6 vs 7), but significantly reduced the amount of Myc-UBXN1 and K63-Ub co-precipitated with FLAG-caspase-4/11 (Lanes 4 vs 5, and 6 vs 7) (Fig.7f). We also noted that free K63-Ub chains bound to FLAG-caspase-11 better than FLAG-APEX-caspase-4, relative to their IP input (Lanes 4 vs 6) (Fig.7f). This difference could be due to the large APEX tag (27 kDα), which may obstruct the interaction between free polyUb and caspase-4. Indeed, relative to their IP input, FLAG-caspase-4 pulled down the similar amount of K48/63-Ub as FLAG-caspase-11 did (IP, Lanes 2 vs 4) (Fig.7g).

Next, we used USP5 (also known as isopeptidase T, IsoT) to specifically digest unanchored polyUb chains without disrupting regular protein-protein interactions (Fig.7e). USP5 obviously reduced the amount of free K48/63-Ub precipitated by FLAG-caspase-4/11 (Beads-bound, Lanes 2 vs 3, and 4 vs 5). This reduction was correlated with the increase of mono-ubiquitin in the USP5 eluate (Fig.7g). Notably, the binding of Myc-UBXN1 to FLAG-caspase-4/11 was also disrupted by USP5. These results demonstrate that UBXN1 mediates unanchored K48/63-Ub binding to caspase-4/11 and stable caspase-4/11 binding to UBXN1 requires unanchored K48/63-Ub.

To see if free ubiquitin chains were involved in the caspase-11 inflammasome *in vivo* during LPS sepsis, we purified total free ubiquitin chains from the spleen and liver. We took advantage of the specificity of USP5 binding to free ployUb and employed a recombinant GST-tagged zinc finger domain (ZnF-UBP, aa 163-291) of USP5 to pull down free ployUb. Indeed, for proof of principle, we detected free K48-Ub, the most abundant polyUb (Supplemental **Fig.S9a**, **b**). The abundances of free K48-Ub chains in the spleen and liver increased after LPS injection intraperitoneally (8 and 20 h), compared to the untreated (0 h). Notably, caspase-11 was precipitated together with free polyUb in a time-dependent manner upon LPS treatment (Supplemental **Fig.S9a**, **b**). Overall, these *in vivo* and in *cellulo* studies support the physical interactions among unanchored K48/63-Ub, UBXN1 and caspase-4 under physiological conditions.

The above results firmly establish the interactions among UBXN1, free unanchored K48/63-Ub and caspase-4. Next, we asked if unanchored K48/63-Ub chains play a functional role in caspase-4/11 activation. Because unanchored polyUb can be derived from *de novo* synthesis by specialized E_2_/E_3_ and deubiquitylation of substrates by numerous DUBs ^35^, it is challenging to genetically deplete intracellular unanchored polyUb. Our knowledge about unanchored polyUb has been largely obtained from the research on USP5 (IsoT) ^35^; however, genetic manipulation of USP5 can alter many cellular activities due to impaired proteasomal protein degradation. To overcome this, we developed a cell-free system for the caspase-4/11 inflammasome. We primed HeLa cells with human IFN-γ, lysed cells by douncing, removed nuclei, large membranes, and debris by centrifuging at 14,000 x g. The cleared lysate was then incubated with WT or mutant LPS of various concentrations (Fig.7h, Supplemental **Fig.S10a**). We noted that the Rd and Re mutants without the outer core and O-antigen more potently activated caspase-4 and GSDMD cleavage than WT and the Ra mutant (Supplemental **Fig.S10b**). Re-LPS-induced GSDMD cleavage was abolished in *UBXN1*^−/−^ and *CASP4*^−/−^ cells, demonstrating that the cell-free system is appropriate for studying the caspase-4 inflammasome (Supplemental **Fig.S10c, d**). We then investigated if USP5 inhibited the caspase-4 inflammasome. On one hand, pretreatment of the WT cell lysate with a recombinant USP5 protein resulted in a notable reduction in Re-LPS -activated caspase-4/11 and GSDMD cleavage (Fig.7i). On the other hand, pharmacological inhibition of endogenous USP5 enhanced the caspase-4 inflammasome (Fig.7j). However, the effect of either the USP5 inhibitor or recombinant USP5 was lost in *UBXN1*^−/−^ cells as compared to WT controls (Fig.7j, k), suggesting that free K48/63-Ub chains promote caspase-4 inflammasome activation in a UBXN1-dependent manner.

### Ternary complex synergistically drives the non-canonical inflammasome activation

**Fig. 8.**
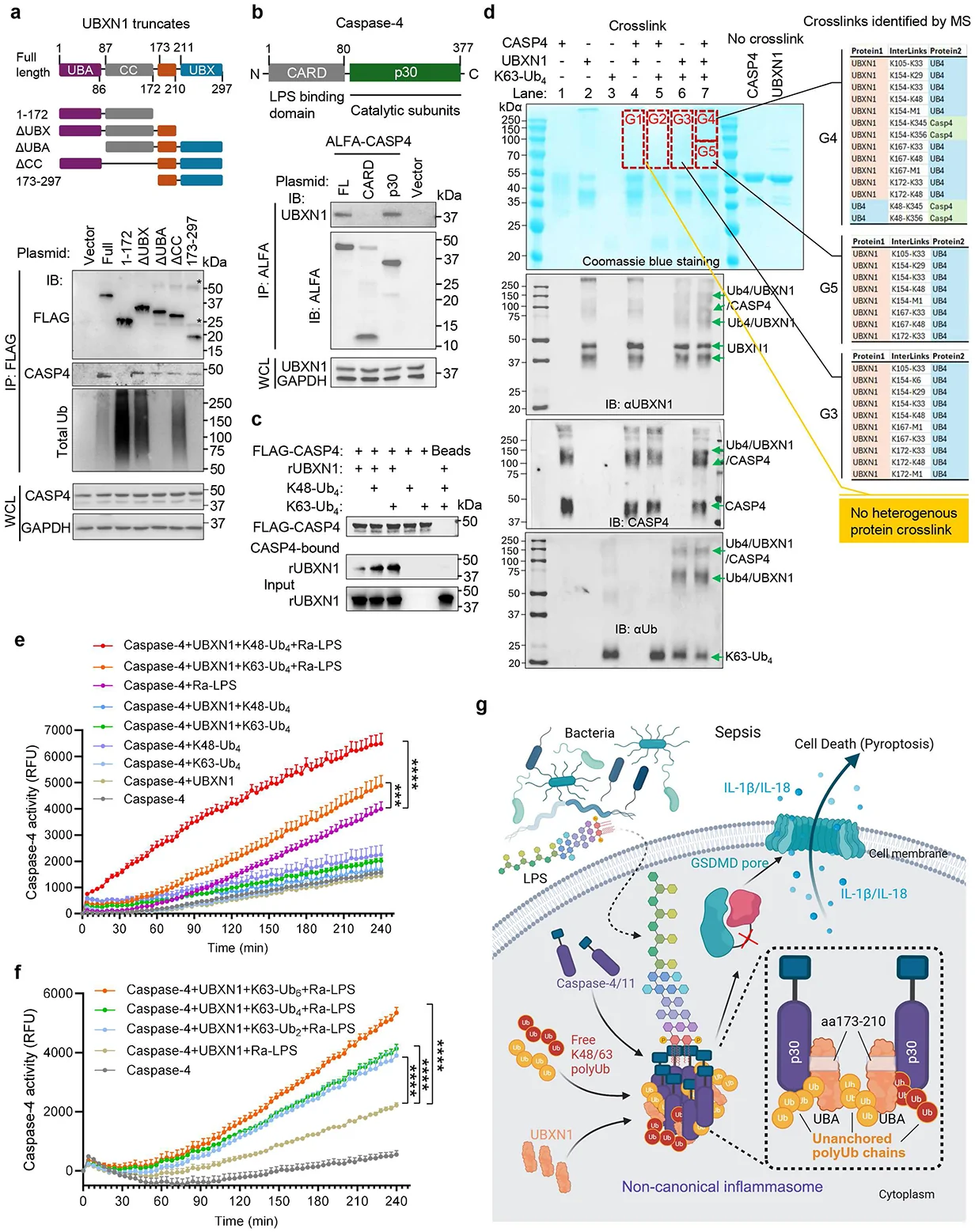
Cooperative action of UBXN1 and unanchored K48/63-Ub on caspase-4. (**a**) Co-IP of FLAG-UBXN1 truncates and endogenous caspase-4 with an anti-FLAG antibody from HeLa cells that were transfected with the indicated plasmids and primed with IFN-γ. (**b**) Co-IP of ALFA-caspase-4 and endogenous UBXN1 with an anti-ALFA antibody from HEK293T cells that were transfected with the indicated plasmids. (**c**) *In vitro* pulldown of purified, HEK293T-derived FLAG-caspase-4 (bound to anti-FLAG magnetic beads) together with *E. coli*-derived 6xHis-UBXN1 (rUBXN1) and *E. coli*-derived K48/K63-Ub_4_. (**d**) *In vitro* crosslinking protein interaction assay with purified recombinant UBXN1, enzymatically inactive mutant caspase-4^C254A^, K63-Ub_4_ of various combinations. Shown are the SDS-PAGE gels stained by Coomassie blue or immunoblotted (IB) with specific antibodies. The green arrows indicate protein monomers and possible complexes. The protein monomers appear smaller and more smeared with the crosslinker BS3 than without. The right panel shows the crosslinks between the indicated proteins identified by crosslink-MS. (**e, f**) *In vitro* caspase-4 activity assay with a fluorogenic substrate, purified insect-derived caspase-4, *E. coli*-derived 6xHis-UBXN1 and K48/63-Ub_n_, and Ra-LPS. Data point, mean + S.E.M., N=3 biological replicates, **p<0.01, ****p<0.0001 by two way-ANOVA with repeated measures, Sidak’s multiple comparisons test. (**g**) Graphical summary of UBXN1 action on caspase-4. UBXN1 interacts with unanchored K48- or K63-Ub chains via its N-terminal UBA domain, and with caspase-4 catalytic domain via its aa 173-210 (between the coiled-coil and UBX domain). Unanchored K48- or K63-Ub chains stabilize UBXN1-caspase-4 interaction. UBXN1 and unanchored K48/63-Ub chain synergistically promote the caspase-4 inflammasome. Created with BioRender.com.

All of the aforementioned results strongly suggest that UBXN1 tethers unanchored K48/63-Ub to caspase-4, thereby promoting inflammasome assembly and activation. We next dissected the ternary polyUb-UBXN1-caspase-4 interaction and mapped the specific functional domains required for complex formation. Deletion of the N-terminal UBA domain (ΔUBA), not the other truncations, completely abolished UBXN1 binding to polyubiquitin chains, suggesting that the UBA domain binds to polyubiquitin chains. The C-terminal amino acid residues (aa) 173-297 (end), aa 87-297 (ΔUBA), ΔCC (1-86, 173-297) and aa 1-210 (ΔUBX) remained capable of binding to caspase-4, while the aa 1-172 was unable, suggesting that the aa 173-210 region mediates UBXN1 interaction with caspase-4 (Fig.8a). The catalytic subunit p30 of caspase-4 was able to bind UBXN1, while the CARD domain could not, suggesting that the p30 mediates caspase-4 interaction with UBXN1 (Fig.8b).

To strengthen the concept that unanchored K48/63-Ub and UBXN1 directly cooperate to regulate the non-canonical inflammasome, we performed *in vitro* protein-protein interaction assays. We first expressed and purified FLAG-caspase-4 from HEK293 cells, then incubated it with *E. coli*-derived recombinant 6xHis-UBXN1 and K48/63-Ub_4_ (Supplemental **Fig.S11a**). FLAG-caspase-4 binding to UBXN1 was significantly enhanced in the presence of either K48-Ub_4_ or K63-Ub_4_ (Fig.8c). We then employed an *in vitro* crosslinking protein interaction assay that stabilizes transient or labile interactions. If a protein complex is formed, bands of high-molecular-weight species will be detected on SDS-PAGE gels and immunoblots. As shown in Fig.8d, incubation of enzymatically inactive mutant caspase-4^C254A^ (derived from insect cells) was incubated with either UBXN1 or K63-Ub_4_ alone did not produce significant high-molecular-weight species, indicating minimal direct interaction under these conditions (Lanes 4 and 5, Fig.8d). Co-incubation of UBXN1 and K63-Ub_4_ resulted in a noticeable reduction of monomeric UBXN1 and K63-Ub_4_ bands, accompanied by the appearance of higher molecular weight species, indicative of complex formation (Lane 6). This is not surprising as UBXN1 contains a UBA domain that directly interacts with polyubiquitin chains. Critically, the addition of caspase-4 further enhanced the formation of polymeric species and reduced the monomeric forms of all three proteins, suggesting that UBXN1, caspase-4, and polyUb chains assemble into a tripartite complex (Lane 7, Fig.8d). Notably, we further identified crosslinking sites (Lys-Lys, Lys-Met) between UBXN1 and K63-Ub_4_, UBXN1 and caspase-4, K63-Ub_4_ and caspase-4 by UPLC-MS/MS (Right panel, Fig.8d).

To investigate the effect of direct binding of UBXN1 and K48/63-Ub to caspase-4 on its activity, we performed an *in vitro* caspase-4 activity assay with a fluorogenic substate. Ra-LPS-stimulated caspase-4 activity was significantly enhanced by UBXN1 and Ub_4_ together (Red and orange vs purple curves, Fig.8e), whereas Ub_4_ alone had no such effect (Supplemental **Fig.S11b**). Intriguingly, K63-Ub chains promoted caspase-4 activity in a length-dependent manner, *i.e*., Ub_6_>Ub_4_>Ub_2_) (Fig.8f), while K11-Ub≧3 had no effect (Supplemental **Fig.S11c**).

Unanchored polyUb chains are synthesized *de novo* by specialized ubiquitin conjugating enzyme (E_2_) and ligase (E_3_) pairs or derived from deubiquitination of substrates ^35^. To see if caspase-4 activation requires *de novo* synthesis of ubiquitin chains, we employed a ubiquitin-activating enzyme E_1_ inhibitor, TAK-243, to stop new synthesis of ubiquitin chains (both unanchored and anchored). TAK-243 significantly reduced total ubiquitination (mostly anchored ubiquitin chains), but did not affect unanchored ubiquitin chains (Supplemental **Fig.S12a**). However, Re-LPS-induced caspase-4 and GSDMD activation was intact in the presence of TAK-243, suggesting that UBXN1 can enrich sufficient existing free K48/63-Ub chains for caspase-4 and does not depend on *de novo* synthesis (Supplemental **Fig.S12b**). We also noted that Re-LPS increased global ubiquitination, but independently of caspase-4 (Supplemental **Fig.S12a, c**). This upregulated ubiquitination is likely due to the ubiquitination of LPS *per se* by RNF213, leading to autophagy ^36^.

## DISCUSSION

Although *in vitro* caspase-4 can be directly activated by LPS, in the complex intracellular environment, efficient activation of caspase-4 may require additional cellular factors ^37^. Guanylate binding proteins (GBPs) could serve as a platform to assemble free cytosolic LPS and caspase-4 ^38^. However, most studies suggest that GBPs assemble on the surface of Gram-negative bacteria into polyvalent signaling platforms for recruiting caspase-4 ^39-41^. In this study, we discover that UBXN1 and unanchored K48/63-Ub chains together promote the caspase-4 inflammasome activation. Mechanistically, UBXN1 directly interfaces with and bridges unanchored K48/63-Ub and caspase-4, forming a tripartite complex, where each bilateral interaction needs strengthening by the third party.

### The mechanism of UBXN1 action during sepsis

UBXN1 has been shown to inhibit RIG-I (retinoic acid-induced gene I)-like receptor (RLR) signaling (the primary viral RNA sensor) ^42^ and NF-κB activity ^26,43^; regulate endoplasmic reticulum-associated degradation (ERAD) positively or negatively ^44-50^; promote the formation of aggresomes ^51^ and mitophagy ^52^. However, all these studies were conducted *in cellulo*, lacking the *in vivo* physiological relevance. With an inducible *Ubxn1* knockout mouse model, we show here that *Ubxn1* deficiency renders animals resistant to caspase-11 inflammasome-dependent LPS sepsis and polymicrobial CLP sepsis ^5-8^, impairs caspase-11 and GSDMD cleavage, reduces inflammasome-dependent and -independent cytokines and tissue damage (Figs.3, 4; Supplemental **Fig.S5**). Consistently, the whole-genome transcriptome analysis reveals a crucial role for UBXN1 in cytokine expression and signaling in splenic macrophages during sepsis (20 h after LPS) (Fig.4d-f, Supplemental **Fig.S6**). Particularly, down-regulated DEGs (*Ubxn1*^−/−^ vs WT) are enriched in chemokines/receptors that mediate immune cell migration and/or activation, including neutrophil (CXCL2, PPBP), monocyte (CCL5), macrophage (CCL22), dendritic cell (CCRL2, XCL1, CCR7), B cell (ACKR3), and T cell (CCL22, CCL5, CXCL10, CXCL11, CXCL16). The upregulated DEGs are predominantly enriched in the cell cycle pathway, consistent with attenuated sepsis in *Ubxn1*^−/−^, because the cell cycle is suppressed during sepsis. These observations suggest that UBXN1 promotes the non-canonical inflammasome, thus inflammatory responses during sepsis. Nonetheless, UBXN1 could also influence sepsis pathogenesis by enhancing mitophagy ^52^ or inhibiting NF-κB activity ^26,43^. However, mitophagy inhibits inflammatory responses and alleviates tissue damage during sepsis ^53^, and NF-κB activity does the opposite ^54^, inconsistent with reduced inflammation in *Ubxn1*^−/−^ mice.

### The role of UBXN1 and unanchored K48/63-Ub in activating caspase-4

Unanchored polyUb chains are synthesized *de novo* by specialized ubiquitin conjugating enzyme (E_2_) and ligase (E_3_) pairs or derived from deubiquitination of substrates ^35^. However, the physiological functions of unanchored polyUb chains have been only recently appreciated. Earlier, unanchored polyUb chains were believed to be toxic to cells, due to inhibition of proteasomes and subsequent accumulation of undesirable proteins ^55-59^. Recent studies, however, have shown their crucial functions in immune pathways and stress responses. For instance, unanchored K63-Ub chains bind to TAB2 [TAK1 (transforming growth factor-beta activated kinase 1)-binding protein 2], leading to activation of the TAK1, IKK (IκB Kinase) complex and NF-κB (nuclear factor κB) in the IL-1 receptor signaling cascade ^60^. K63-Ub decorated with Met1-Ub (namely linear polyUb) bind to NEMO (IKKγ/NF-κB essential modulator), resulting in formation of the A20-NEMO complex and termination of NF-κB signaling in the Toll-like receptors (TLRs) and TNFR (tumor necrosis factor receptor) pathways, respectively ^61-63^. Unanchored K63-Ub chains bind to RLRs to promote the immune response to RNA viruses ^64,65^. Unanchored K48-Ub chains bind to IKK-ε to activate itself and STAT1 phosphorylation (signal transducer and activator of transcription 1), leading to interferon (IFN) expression and signaling, respectively ^66^. Our study extends the functions of free polyUb to inflammasomes. Our *in vitro* studies with purified proteins suggest that UBXN1 directly interacts with free K48/63-Ub chains strongly, while the interaction between UBXN1 and caspase-4, K48/63-Ub and caspase-4, is relatively weak (Fig.8d). Notably, each bilateral binding can be strengthened by the third party, suggesting that all three proteins interface with each other to stabilize the protein complex (Fig.7a, f, g; Fig.8c, d), thus facilitating caspase-4 activation by LPS (Fig.8e, f; Supplemental **Fig.S11b**, **c**). This mode of interaction, where both UBXN1 and free polyUb are simultaneously required, may minimize spontaneous activation by abundant unanchored polyUb chains *per se* in cells. For example, a cell may contain ~6000 unanchored K63-Ub molecules at steady-state^64^ and free K48-Ub chains are much more abundant than K63-Ub. Nonetheless, accumulation of unanchored ubiquitin chains in the vicinity of a target may be necessary for efficient binding and activation of the target. This can be achieved by bringing a specialized E_3_ ligase like TRIM25 (tripartite motif containing 25) or TRAF6 (TNF receptor associated factor 6) to provide *de novo* synthesis of unanchored polyUb to RLRs ^64^ or TAB2 ^60^, respectively. Alternatively, a ubiquitin chain-binding protein like UBXN1 can enrich and stabilize existing unanchored polyUb chains around a target like caspase-4. This is further substantiated by the result showing that the caspase-4 inflammasome is independent of *de novo* synthesis of ubiquitin chains, regardless of anchored or unanchored (Supplemental **Fig.S12**).

Although independently of specific ubiquitination enzymes, caspase-4 still selectively binds to K48-Ub and K63-Ub chains via UBXN1 (Fig.6d; Supplemental **Fig.S7**). This specificity may be related to the structural difference of different polyUb linkages. Indeed, unanchored K11-Ub chains are unable to synergize with UBXN1 to activate caspase-4 *in vitro* (Supplemental **Fig.S11c**). Moreover, cellular free K48-Ub chains are way more abundant than the other free polyUb chains and bind to UBXN1 more strongly than K63-Ub ^34^, thus may be the dominant free polyUb in activating caspase-4 under physiological conditions.

### Limitations of the study

In sum, our data strongly supports a positive role for unanchored K48/63-Ub chains and UBXN1 in the caspase-4 inflammasome and pathogenesis of polymicrobial sepsis that closely resembles the progression and characteristics of human sepsis (Fig.8g) ^28^. Notably, UBXN1 expression is significantly increased in patients experiencing life-threatening sepsis ^67^. Therefore, UBXN1 could be a potential drug target for human septicemia. However, we cannot exclude that UBXN1 may contribute to sepsis pathogenesis *in vivo* independently of the caspase-4 inflammasome, *e.g*., chemokine signaling, as suggested by the RNA-seq data. Moreover, because free K48/63-Ub binding to caspase-4 is reliant on UBXN1, it remains challenging to pinpoint the caspase-4 domain that directly interfaces with free K48/63-Ub chains. Tissue specific *Ubxn1* knockout animal models and structural analysis of the UBXN1/K48/63-Ub/caspase-4 complex may be helpful to address these outstanding questions.

## MATERIALS AND METHODS

### Mouse models

All the animal procedures were approved by the Institutional Animal Care and Use Committee at UConn Health adhering to federal and state laws. We applied a CRISPR-Cas9 method to generate a LoxP-flanked *Ubxn1*^flox/flox^ mouse line at Yale Genome Editing Center (New Haven, CT 06520, USA). Briefly, a *loxP* sequence was inserted into the intron between Exons 2 and 3 (5’-*loxP*) and the intron between Exons 9 and 10 (3’-*loxP*) of Ubxn1 gene in C57BL/6 embryonic stem (ES) cells. Appropriately modified ES were injected into C57BL/6 mice. The *Ubxn1*^flox/flox^ mouse line was crossed with a tamoxifen inducible Rosa26-CreERT2 line (Stock # 008463, The Jackson Laboratory, Bar Harbor, ME 04609, USA) to generate *ERT2*–Cre^+^ *Ubxn1*^flox/flox^ mice. Genotyping was performed with genomic DNA and Choice Taq Blue MasterMix (Cat# CB4065-8, Denville Scientific, Holliston, MA 01746, USA) under the following PCR conditions: initial denaturing at 95 °C for 2 min, 34 cycles of amplification at 94 °C for 1 min, 60 °C for 30 sec, 72 °C for 30 sec, and then final extension at 72 °C for 7 min. The primers for genotyping 5’-loxp were forward (F): 5’-ATCGAGATGGGCTTTCCCAG-3’ and reverse (R): 5’-GTAGGCAGGGGTTGGTTAGC-3’. The PCR products were 303 bp (mutant allele) and 267 bp (wildtype WT allele). The primers for genotyping 3’-loxp were F: 5’-CTGTGCAGTTGCTCAGTGG-3’ and R: 5’-TCAGCTGGGACACTTCTTGG-3’. The PCR products were 330 bp (mutant allele) and 294 bp (WT allele). The Cre primers were common F: 5’ AAGGGAGCTGCAGTGGAGTA, WT-R: 5’-CCGAAAATCTGTGGGAAGTC, and mutant R: 5’-CGGTTATTCAACTTGCACCA. The PCR resulted in a product of 297 bp (WT allele) and 450 bp (Cre), respectively. To induce global *Ubxn1* knockout, 1 mg of tamoxifen (dissolved in corn oil) was administered to each mouse (>6 weeks old) every other day for five times in total. *ERT2*–Cre^+^ *Ubxn1*^flox/flox^ mice treated with corn oil served as the wild-type control (*Ubxn1*^+/+^). The mice then rested for at least two weeks to allow for complete tamoxifen clearance before any further experimentation. The *Casp11*^−/−^ mouse line originated from the Dixit lab ^68^.

### Reagents and antibodies

The antibodies against GAPDH (Cat# 60004-1-Ig, 1:2000), UBXN1 (Cat# 16135-1-AP, 1:1000), UBXN6 (Cat# 14706-1-AP, 1:1000), GBP1 (Cat# 15303-1-AP, 1:1000) were purchased from Proteintech Group (Rosemont, IL 60018, USA). The antibodies against caspase-4 (Cat# 4450S, 1:1000), GSDMD (Cat# 39754S, 1:1000), cleaved GSDMD (Asp275) (Cat# 36425S, 1:500), cleaved GSDMD (Asp276) (Cat# 10137S, 1:1000), NLRP3 (Cat# 15101S, 1:1000), Tubulin (Cat# 2144S, 1:2000), Actin (Cat#4967S, 1:2000), UBXN3B (Cat# 34945S, 1:1000), K63–linkage specific polyubiquitin (Clone D7A11, Cat# 5621S,1:500), K48-linkage specific polyubiquitin (Clone D9D5, Cat# 12805S, 1:1000), phospho-Jak1 (Tyr1034/1035) (Cat# 74129S, 1:1000), phospho-Stat1 (Tyr701) (Cat# 9167S, 1:1000), Myc-Tag (Cat# 2276S, 1:2000) and HA-Tag (Clone C29F4, Cat# 3724S, 1:1000) antibodies were from Cell Signaling Technology (Danvers, MA 01923, USA). Caspase-1 (Cat# ab179515, 1:1000), caspase-11 (Cat# ab180673, 1:1000), GSDMD (Cat# ab209845, 1:1000) antibodies and recombinant human USP5 protein (active, a.k.a IsoT, Cat# ab269124) were from Abcam (Waltham, MA 02453, USA). The mouse anti-Myc antibody (Clone 9E10, Cat# TA150121, 1:1000) was obtained from OriGene Technologies (Rockville, MD 20850, USA). The antibodies against HUWE1 (Cat# A20708, 1:1000), STUB1 (Cat# A11751, 1:1000), K29-linkage specific polyubiquitin (Cat# A18198, 1:500), K33-linkage specific polyubiquitin (Cat# A18199, 1:500) were purchased from ABclonal Technology (Woburn, MA 01801, USA). The anti–FLAG M2 magnetic beads (Clone M2, Cat# M8823), monoclonal anti–FLAG M2 antibody (Cat# F1804, 1:2000), lipopolysaccharides (*E. coli* O111:B4, Cat# L3024), LPS-Ra mutant (*E. coli* EH100, Cat# L9641), LPS-Rd mutant (*E. coli* F583, Cat# L6893), LPS-FITC conjugate (*E. coli* O111:B4, Cat# F3665), biotin (Cat# B4501), nigericin (Cat# N7143), ATP (Cat# A6419) and tamoxifen (Cat# T5648) were obtained from Sigma-Aldrich (St. Louis, MO 63178, USA). LPS-Re mutant (Lipid A, *E. coli* R515, Cat# ALX-581-007-L001) was from Enzo Biochem (Farmingdale, NY 11735, USA). Recombinant mouse (Cat# 485-MI-100) and human (Cat# 285-IF-100) INF-γ proteins, recombinant human INF-β were obtained from R&D Systems (Minneapolis, MN 55413, USA). The anti-Myc magnetic beads (Clone 9E10, Cat# 88842), donkey anti-mouse Alexa Fluor^™^ Plus 488 (Cat# A32766, 1:200), donkey anti-rabbit Alexa Fluor^™^ Plus 594 (Cat# A32754, 1:200), ubiquitin polyclonal antibody (Cat# 1859660, 1:1000), DAPI (Cat# 62248, 1:1000), Pierce^™^ High Capacity Endotoxin Removal Spin Columns (Cat# 88273), PureLink^™^ Genomic DNA Mini Kit (Cat# K182002), HiPure Plasmid Filter Midiprep Kit (Cat# K210014), and Quick Plasmid Miniprep Kit (Cat# K210010) were from ThermoFisher Scientific (Waltham, MA 02451, USA). K48-linked Ub_4_ (Cat# SBB- UP0070) and K63-linked Ub_4_ (Cat# SBB-UP0073) were products of South Bay Bio (San Jose, CA 95138, USA). K63-linked polyubiquitin (Ub_2-7_) (Cat# UBI-60-0109-100) was from Cosmo Bio (Carlsbad, CA 92010, USA). The TransIT-X2 Dynamic Delivery System (Cat# MIR6005) was from Mirus Bio LLC (Madison, WI 53719, USA). Biotinylated LPS (*E. coli* 0111: B4, Cat# tIrl-Ipsbiot) and flagellin (Cat# vac-fla) were products of InvivoGen (San Diego, CA 92121, USA). Streptavidin Magnetic Beads (Cat# S1420S) was a product of New England Biolabs (Ipswich, MA 01938, USA). The Unconjugated (Free) Polyubiquitin Chain Capture Kit (Cat# J4420), mono-ubiquitin (Ub_1_) (Cat# E1100), K63-Ub_2_ (Cat# E2100), K63-Ub_4_ (Cat# E2300), K63-Ub_6_ (Cat# E2500) and K11-Ub_>3_ (Cat# E3300) were from UBPBIO (Dallas, TX 75252, USA). ALFA selector PE (Cat# N1510; including ALFA peptide N1520) and sdAb anti-ALFA unconjugated (Cat# N1505) were obtained from NanoTag Biotechnologies (Rudolf-Wissell-Straße 28a, 37079 Göttingen, Germany).

### Plasmids

The HA-Ub (WT), HA-K6Ub (all the other K are mutated to R), HA-K11Ub, HA-K27Ub, HA-K29Ub, HA-K33Ub, HA-K48Ub,and HA-K63Ub plasmids were kind gifts of Dr. Rongtuan Lin ^69^. The FLAG-APEX-CASP4, ALFA-CASP4, ALFA -CARD of CASP4, ALFA-p30 of CASP4 and FLAG-CASP1 (human) plasmids were kind gifts from Dr. Vijay A. Rathinam ^70^. The FLAG-CASP4 plasmid was a kind gift from Dr. Jianbin Ruan. The FLAG-Casp11 plasmid (Cat# 21145) was a kind gift from Junying Yuan ^71^; FLAG-CASP2 (Cat# 11811) and FLAG-CASP7 (Cat# 11815) ^72^ were kind gifts from Guy Salvesen via Addgene. The Myc-UBXN1 and Myc-UBXN3B plasmids were described previously ^25,42^.

### Murine models of sepsis

Sepsis was induced by LPS or cecal ligation and puncture (CLP) as described previously ^73,74^. For the CLP model, age- and sex-matched mice were subjected to fasting for 14 h. The mice were anesthetized with ketamine /xylazine and then fixed gently on a warm pad. The animal abdominal skin was disinfected with 70 % ethanol, and a small (~1 cm) longitudinal skin incision was made left to midline. The cecum was ligated at 1.5 cm from the end and perforated (through-and-through puncture) by 5 times near the ligation site with a 21G needle. The ligated cecum was squeezed to confirm patency and extrusion of feces. The cecum was then repositioned into the peritoneal cavity and the abdominal musculature was closed. For the sham group, the similar surgical procedures were performed but without ligation and puncture of the cecum. For sepsis induced by LPS, age- and sex-matched mice were injected intraperitoneally (*i.p*.) with 8, 10 or 12 mg/kg of *E. coli* O 111:B4-derived LPS unless otherwise indicated. Mice injected with the same volume of sterile phosphate buffered saline (PBS) were considered as mock controls.

### Cell culture and isolation of peritoneal or bone marrow-derived macrophages (BMDMs)

HeLa (Adenocarcinoma epithelial cell, Cat# CCL-2), HEK293T cells (human embryonic kidney, Cat# CRL–3216) and L929 cells (mouse fibroblast cells, Cat# CCL-1) were purchased from the American Type Culture Collection (ATCC) (Manassas, VA 20110, USA). HEK293T, HeLa and L929 cells were grown in Dulbecco's modified Eagle's medium (DMEM, Corning, Cat#10-013-CV) supplemented with 10% fetal bovine serum (FBS, Gibco^™^, Cat#A52567-01) and 1% antibiotics/antimycotics (ThermoFisher Scientific, USA). These cell lines are not listed in the database of commonly misidentified cell lines maintained by ICLAC and have not been authenticated in our hands. They are routinely treated with MycoZAP (Lonza, Switzerland) and tested for mycoplasma contamination in our hands.

Bone marrow cells were isolated and differentiated into macrophages (BMDM) using an established method ^75^. Briefly, bone marrow cells were differentiated in L929–conditioned medium (RPMI 1640, 20% FBS, 30% L929 culture medium, 1x antibiotics/antimycotics) in a 10–cm Petri dish at 37 °C, 5% CO_2_ for 5–7 days, with a change of medium every 2 days. Attached BMDMs were dislodged by incubating in ice-cold PBS for 30 min followed by pipetting. The BMDMs were cultured in plating medium (RPMI 1640, 10% FBS, 5% L929-conditioned medium, 1x antibiotics / antimycotics) in tissue culture treated plates.

For isolation of peritoneal macrophages, mice were injected intraperitoneally with 5 ml of 3% (w/v) autoclaved Brewer thioglycollate medium. After 3 days, the mice were euthanized and injected 5 ml of ice-cold PBS into the peritoneal cavity. The abdomen was massaged gently for 2 min to dissociate cells. The peritoneal fluid was then collected using a sterile syringe with 25G needle and transferred to a 15 mL tube on ice. The fluid was filtered through a 70 μm cell strainer to remove tissue debris, and centrifuged at 500xg, 4°C for 5 min. The pelleted cells were then resuspended in complete DMEM with 10% FBS and cultured in cell culture plates at the desired density for 2 h at 37 °C, 5% CO_2_. Non-adherent cells were gently washed off with PBS and the remaining attached cells were macrophages.

### Generation of gene knockout cell lines using CRISPR-Cas9

Knockout of *UBXN1*, *STUB1*, *HUWE1* and *CASP4* in human cell lines by CRISPR-Cas9 was performed according to our previous study ^75^. Briefly, pre-designed, gene unique guide (g) RNAs (Integrated DNA Technologies, Coralville, lowa 52241, USA) were subcloned into the lentiCRISPR-V2 vector ^76^ and correct insertion was confirmed by DNA sequencing. To generate lentiviral particles, each gRNA vector was transfected into HEK293T cells together with packaging plasmids pCMV-VSV-G ^77^ and psPAX2 (Cat#12259 from the Didier Trono lab via Addgene). A half of the cell culture medium was replaced with fresh medium at 24 h and viral particles were collected at 48-72 h after transfection. The cell culture supernatant (viral particles) was cleared by brief centrifugation at 1000 rpm for 5 min. Target cells at ~50% confluence in a 6-well plate were transduced with 1 mL of each lentiviral preparation and 8 μg/mL polybrene for 24 h and selected with 1 μg/mL of puromycin in growth media for 5 days. The knockout efficiency in each cell line was validated by immunoblotting. The sequences for gRNAs are *UBXN1*-#1-F: CACCGTCTAAAGGCTCGTCCACATC and R: AAACGATGTGGACGAGCCTTTAGAC, *UBXN1*-#2-F: CACCGACACTGGTCATACTCCCGCT and R: AAACAGCGGGAGTATGACCAGTGTC, *STUB1*-F: CACCGGGCCGTGTATTACACCAACC and R: AAACGGTTGGTGTAATACACGGCC, *HUWE1*- F: CACCGTTCCATCGAAGCGGTCCAAC and R: AAACGTTGGACCGCTTCGATGGAA, *CASP4*-F: CACCGGAGAAACAACCGCACACGCC and R: AAACGGCGTGTGCGGTTGTTTCTCC.

### Flow cytometry

Flow cytometry was performed essentially according to an established method ^24^. Blood was collected from anesthetized mice and red blood cells were lysed three times with an RBC lysis buffer (Cat. # 42030, BioLegend, San Diego, CA 92121, USA). The remaining white blood cells were suspended in FACS buffer and stained for 30 min at 4 °C with the antibody cocktails of APC-Fire 750-anti CD11b (Cat. # 101261, clone M1/70), Alexa Fluor 700-anti Ly-6G (Cat. # 127621, clone 1A8), PE-Dazzle 594-anti CD3 epsilon (Cat. # 100347, clone 145-2C11), Brilliant Violet 711-anti CD115 (Cat. # 135515, clone AFS98), Zombie UV (Cat. # 423107), PE-Cy7-anti CD45 (Cat. # 103113, clone 30-F11), APC-anti CD19 (Cat. # 152410, clone 1D3/CD19) (BioLegend). After staining and washing, the cells were fixed with 4% paraformaldehyde and counted on a Becton–Dickinson FACS ARIA II, CyAn advanced digital processor (ADP). The data was analyzed using FlowJo software. Among CD45+ cells, CD11b+ Ly6G+ cells were classified as neutrophils, Ly6G− CD11b+ CD115+ as monocytes, CD3+ as total T cells, CD19+ as B cells.

### Induction of the inflammasomes

For activating the non-canonical inflammasome, HeLa cells were primed with 10 ng/mL of human IFN-γ for 12 h, and then transfected with 1 μg/mL of LPS (*E. coli* O111:B4) (or Bio-LPS, FITC-LPS for immunofluorescence) using TransIT-X2 Dynamic Delivery System, and incubated further for 5 h. BMDMs were primed with murine IFN-γ (10 ng/mL) for 3 h and then transfected with LPS (1 μg/mL) for 16 h. The non-canonical inflammasome in BMDMs was also activated with 10 μg/mL of *E. coli*-derived outer membrane vesicles (OMVs) (InvivoGen, Cat# tlrl-omv-1) for 16 h. For caspase-4 overexpression-induced inflammasome response, HeLa cells were treated with human 10 ng/mL of IFN-γ for 12 h and then transfected with FLAG-CASP4 and/or Myc-UBXN1 plasmids for 24 h. To stimulate the canonical NLRP3 inflammasome, primary BMDMs were primed with LPS (1 μg/mL) for 3 h and then stimulated with ATP (5 mM) or nigericin (10 μg/mL) for 30 min. To stimulate the canonical NLRC4 inflammasome, primary BMDMs were primed with LPS (1 μg/mL) for 3 h and then transfected with flagellin (20 μg/mL) for 4 h.

### Immunoprecipitation (IP)

To immunoprecipitate FLAG-, Myc- or ALFA-tagged recombinant proteins, HEK293T/HeLa cells were transfected with expression plasmids using TransIT-X2 Dynamic Delivery System for 24 h. In some cases, cells were then primed with human IFN-γ for 12 h and/or transfected with 1 μg/mL of LPS for 5h. Cells were lyzed in an IP lysis buffer (150 mM NaCl, 50 mM Tris, pH 7.5, 1 mM EDTA, 0.5% NP40, 10% glycerol, protease inhibitors) on ice for 15 min. The lysate was centrifuged at 15,000 g for 10 min in a microcentrifuge to remove cell debris, then incubated with 30 μL of anti-FLAG^®^ M2 (Cat # M8823, Millipore), anti-c-Myc magnetic beads (50% v/v) (Cat # 88842, ThermoFisher), or ALFA-Select nanobody (Cat# N1510, NanoTag Biotechnologies), with gentle rotating overnight at 4 °C. The beads were washed 3 times with 1x or 5x Tris-buffered saline (TBS) and bound proteins were eluted with a 3x FLAG, Myc or ALFA peptide.

To immunoprecipitate endogenous caspase-4 or UBXN1, ~ 8x10^6^ HeLa cells were primed with human IFN-γ for 12 h and then transfected with 1 μg/mL of LPS for 3, 6, and 9 h. Cells were lyzed in a lysis buffer (50 mM Tris-HCl pH 7.4, 150 mM NaCl, 1 mM EDTA, 1% Triton-100, 1x protease inhibitors) on ice for 15 min. The lysate was centrifuged at 15,000 g for 10 min in a microcentrifuge to remove cell debris at 4°C, then incubated with 2 μg of an anti-caspase-4 (Cat# 42264S, Cell Signaling Technology), anti-UBXN1 (Cat# 16135-1-AP, Proteintech) antibody, or control IgG, with gentle rotating at 4 °C overnight. Then, 30 μL of protein A/G magnetic beads (Cat# 88802, ThermoFisher) were added and incubated with gentle rotating at room temperature for 1 h. The beads were spun down by brief centrifugation and washed 3 times with a wash buffer (50 mM Tris-HCl pH 7.4, 300 mM NaCl, 1 mM EDTA, 0.05% Tween-20). The beads were then incubated with 1XSDS-PAGE reducing sample buffer (50mM Tris-HCl, 2% SDS, 100mM DTT, 10% Glycerol, 0.1% bromophenol blue dye, pH6.8) at room temperature for 10 min to elute bound proteins. The eluants were heated at 98°C for 5 min and subjected to immunoblotting.

### Tandem mass spectrometry

After immunoprecipitation, the beads-bound proteins were digested with trypsin, and the resultant peptides were analyzed by ultra performance liquid chromatography-tandem mass spectrometer (UPLC-MS/MS) at the UConn Proteomics & Metabolomics Facility. Scaffold 5 (Proteome Software, Portland, OR 97219, USA) was employed to calculate protein abundances. Results were expressed as Log_2_ fold change in average precursor intensity (API), *i.e*., Log_2_ (caspase-4 bound/vector). The frequencies of ubiquitinated lysine residues in caspase-4 were expressed as percentage (%) of di-glycine-modified peptides of total peptides counts containing the corresponding lysine residue.

### Streptavidin-biotin pulldown

~ 8x10^6^ HeLa cells in a 10-cm dish were primed with 10 ng/ml IFN-γ for 12 h and then transfected with 1 μg/mL of biotin or biotin-conjugated LPS for 5 h. Cells were lysed in a nonionic detergent buffer (50 mM Tris-HCl pH7.4, 150 mM NaCl, 5 mM MgCl2, 0.1% Triton X-100) on ice for 15 min. The lysates were centrifuged for 10 min at 4 °C, 6000 g to remove cell debris. The supernatants were incubated with streptavidin magnetic beads (Cat # S1420S, New England Biolabs) with gently rotating at room temperature for 2 h. The beads were washed 3 times in wash buffer (20 mM Tris-HCl, pH7.5, 0.5 M NaCl, 1 mM EDTA), then boiled in a SDS-PAGE sample loading buffer for 10 min to elute biotin-LPS and bound proteins.

### Unconjugated (free) polyubiquitin chain capture

This assay is based on the specific binding of USP5 via its N-terminal ZnF-UBP domain to the C-terminal diglycine motif of free polyUb chains. A recombinant GST (glutathione S-transferase)-ZnF-UBP protein (Cat# J4420, UBPBio) was used to capture free polyUb chains from tissue lysates. Briefly, tissues were homogenized in a lysis buffer (20 mM Tris, pH 7.2, 150 mM NaCl, 2 mM 2-mercaptetanol, 5 mM iodoacetamine, 1x protease inhibitor and 10% glycerol) (1 mL/g tissue) with a handheld tissue grinder on ice. Tissue cells were lyzed by snap freezing-thawing 4 times and the resulting lysates were centrifuged at 15,000 rpm in a microcentrifuge for 10 min at 4°C. Approximately 10 mg total protein lysates were incubated with 0.2 mg/mL of GST or GST-ZnF-UBP and 75 μL of glutathione agarose resin (net resin volume. The resin was washed three times with the lysis buffer before use), gently rotating for 3 h at 4°C. After incubation, the agarose resin was spun down at 500 g for 5 min, washed with 1.5 mL of the lysis buffer 3 times. GST/GST-ZnF-UBP and its bound proteins were eluted with 100 μL of 10 mM glutathione (prepared in TBS, pH 7.0).

### Activation of caspase-4/11 by LPS in a cell-free system

Approximately 8x10^6^ HeLa cells in a 10-cm dish were primed with 20 ng/ml of human IFN-γ overnight and washed twice with ice-cold PBS. The cells from 2 dishes were scraped off in 500 μL of suspension buffer (150 mM NaCl, 50 mM Tris-HCl, 1 mM EDTA, 10% glycerol, 1x protease inhibitors). The cell suspension was forced rapidly through a 25G needle 45~50 times to lyze cells, then centrifuged for 15 min at 14,000 g, 4 °C to remove debris. For a standard cell-free reaction, 10 μL of LPS (diluted in PBS) and 10 μL of the above lysate were incubated together for 4 h at 37 °C. When necessary, 10 μL of cell lysate were incubated with 2 μL of recombinant USP5 (50 ng/μL) or USP5 inhibitor (USP5-IN-1,20 μM) for 1 h at 30 °C. Then 10 μL of Re-LPS (250 μg/mL) and 3 μL of USP5 (50 ng/μL) or USP5-IN-1 (20 μM) were added into the reaction, and incubated for 4 h at 37 °C. The reaction was stopped with 8 μL of 4x Laemmli protein sample buffer (Bio-Rad, Cat# #1610747).

### *In vitro* assay for caspase-4 enzymatic activity

6xHis-caspase-4 and 6xHis-UBXN1 were expressed and purified from Sf9 cells and *E. coli*, respectively. Unanchored polyUb chains from commercial sources were enzymatically conjugated with a specific E2 and *E. coli*-derived human mono-Ub, then purified via liquid chromatography. Endotoxins were removed from UBXN1using a Pierce^™^ High-Capacity Endotoxin Removal Spin Columns kit (Cat# 88273, ThermoFisher). Recombinant caspase-4 (1 μM), UBXN1 (1 μM), K48/K63-Ub_n_ (1 μM) and Ra-LPS (0.5 μM) were mixed (in various combinations) in HEPES buffer (25 mM HEPES, 150 mM NaCl, 2 mM DTT, 0.5 mM EDTA, pH7.5) on ice. In a 384-well black plate, 30 μL of Ac-LEVD-AMC (a fluorogenic caspase-4 substrate, 5 mM in DMSO, Enzo, Cat# ALX-260-083-M001) were loaded into each well, followed by 30 μL of the above reaction mix. The fluorescence intensity was recorded continuously every 4 min by a fluorescence microplate reader. The concentration of each component was optimized in our preliminary studies.

### *In vitro* assay for protein-protein interactions

Two *in vitro* assays were employed to assess direct UBXN1/caspase-4/ polyUb interactions. In the first experiment, a FLAG-CASP4 or FLAG vector (negative control) plasmid was transfected into HEK293T cells (four 10-cm dishes) for 24 h. FLAG-caspase-4 or FLAG protein was purified with 160 μL of anti-FLAG M2 magnetic beads as described in the Immunoprecipitation section above. The beads were then split equally into 5 aliquots, incubated with *E.coli*-derived recombinant human UBXN1 (4 μM) and K48-Ub_4_ (1μM) or K63- Ub_4_ (1μM) in HEPES buffer (25 mM HEPES, 150 mM NaCl, 2 mM DTT, 0.5 mM EDTA pH7.5), gently rotating at 37 °C for 4 h. The reaction mixes were centrifuged briefly to separate the beads (bound fraction) from liquid (unbound fraction). The beads were washed, and bound proteins were eluted as described in the Immunoprecipitation section above. In the second experiment, an *in vitro* crosslinking protein interaction assay was performed to detect transient interactions. Various combinations of Sf9 cell-derived 6xHis- enzymatically inactive mutant caspase-4^C254A^ (10 μM), *E.coli*-derived 6xHis-UBXN1 (10μM) and K63-Ub_4_ (10 μM) were incubated at room temperature for 2 h, then with BS3 (bis(sulfosuccinimidyl)suberate) (Cat# 21580, ThermoFisher) (2.5 mM) on ice for 1 hr. The cross-linking reaction was quenched with 100 mM Tris-HCl (pH 8.0) at room temperature for 15 min. The reaction mixes were subjected to SDS-PAGE then immunoblotting or Coomassie blue staining and UPLC-MS/MS to identify BS3-crosslinked peptides (N-terminus or Lys focused). The gel bands were destained then subjected to Cys reduction and alkylation using dithiothreitol and iodoacetamide, respectively. The embedded proteins were then digested overnight (16 h) using sequencing grade modified trypsin. After the digestion was complete, the peptides were extracted using alternating washes with aqueous and nonpolar solvents, dried to completion, resuspended in 0.1% formic acid in water and quantified via A280 measurements on a Nanodrop spectrophotometer. The samples were all diluted to equal concentrations for UPLC-MS/MS and analyzed using the same 1 h data-dependent acquisition (DDA) UPLC-MS/MS acquisition method on a Q Exactive HF mass spectrometer.

### Quantification of lactate dehydrogenase and cytokines

Lactate dehydrogenase (LDH) release into the cell culture medium is an indicator of cell death. LDH was quantified with the Cytotoxicity Detection Kit^PLUS^ (LDH) kit (Cat# 04744926001, Roche, Indianapolis, Indiana, USA) according to the manufacturer’s protocol. The release of IL-1β or IL-18 into cell culture supernatants was quantified by enzyme-linked immunosorbent assay (ELISA) (Cat# DY401-05, DY318-05, R&D Systems). The concentrations of cytokines and growth factors in mouse sera, peritoneal lavages and culture medium were measured with a LEGENDplex^™^ bead-based multiplex ELISA kit (BioLegend). The procedures were performed exactly according to the product manual. Briefly, 25 μL of samples or standards were mixed with antibody-coated premix microbeads in a filter–bottom microplate and incubated at room temperature for 2 h with vigorous shaking at 500 g. The beads were washed twice to remove unbound analytes. Then 25 μL of detection antibody were added to each microplate well, and the plate was incubated at room temperature for 1 h with vigorous shaking at 500 g. 25 μL of the SA–PE reagent was then added directly to each well, and the plate was incubated at room temperature for 30 min with vigorous shaking at 500 g. After two washes, the beads were resuspended in 1x wash buffer and transferred to a 96-well microplate for reading on a BIORAD ZE5 cell analyzer. The concentration of each analyte was calculated with the standards using the LEGENDPlex data analysis software.

### Immunofluorescence microscopy

Cells were grown on chambered coverslips (Cat # 80826, Ibidi, Gräfelfing, Germany) and stimulated with IFN-γ/LPS as necessary. Cells were washed with ice-cold PBS twice, fixed with 4% paraformaldehyde (PFA) for 15 min at room temperature. The cells were then washed twice with ice-cold PBS, incubating 5 min per wash, then permeabilized with 0.25% Triton X-100 in PBS (PBST) for 10 min at room temperature. The cells were washed twice and blocked with a blocking buffer (10% goat serum, 0.1% bovine serum albumin, 0.3M glycine in PBST) for 1 h at room temperature. Primary antibodies (anti-caspase-4/11, 1:200, R&D Cat# NB120-10454; anti-UBXN1, 1:200, Proteintech Cat# 16135-1-AP) were diluted in an antibody dilution buffer (1% BSA, 0.3M glycine in PBST) and incubated with cells overnight at 4 °C. The cells were washed 3 times in PBS for 5 min per wash and then incubated with an Alexa Flour^™^ -conjugated secondary antibody (ThermoFisher, 1:200 dilution) for 1 h in the dark at room temperature. The cells were washed 3 times and stained for the nuclei with 4',6-diamidino-2-phenylindole (DAPI) (ThermoFisher, Cat # D1306, 1:1000 dilution) for 10 min in the dark at room temperature. Finally, the cells were washed once with PBS and imaged with a Zeiss 880 laser scanning confocal microscope (objective 63 x oil lens). The images were processed with the Zen 2.3 microscopy software (Zeiss Group, Germany).

### SDS-PAGE and immunoblotting

Immunoblotting was performed using a standard method in our previous studies ^78^. In brief, protein samples were resolved by (SDS-PAGE) and transferred to a nitrocellulose membrane. The membrane was blocked with 5% (w/v) skim milk, incubated with a primary antibody [diluted in 5% bovine serum albumin (BSA)]. An anti-rabbit IgG, horseradish peroxidase (HRP)-conjugated secondary antibody (Cat#7074, Cell Signaling Technology) or anti-mouse IgG, HRP-conjugated secondary antibody (Cat#7076) was used at a dilution of 1:5000 or 1:10000 in 5% (w/v) skim milk. Immunoblots were developed with an enhanced chemiluminescent (ECL) substrate (Cat# 32106, ThermoFisher Scientific) or an ultra-sensitive Lumigen ECL Ultra substrate for low-femtogram-level detection (Cat# TMA-100, Lumigen, Southfield, MI 48033, USA). Protein bands were detected and acquired by a ChemiDoc MP Imager (Bio-Rad), and semi-quantified by Image J.

### Extraction of total RNA and RT-qPCR

Quantitative reverse transcription PCR was performed as in a previous study ^79^. Cells or tissues were collected in 350 μL of lysis buffer (RNApure Tissue & Cell Kit, CoWin Biosciences, Cambridge, MA 02138, USA), containing 1% 2-mercaptoethanol. Total cellular RNA was extracted according to the product manual. Reverse transcription of RNA into complementary DNA (cDNA) was performed using the PrimeScript^™^ RT reagent Kit (Cat# RR037A, TaKara Bio, San Jose, CA 95131). Quantitative PCR (qPCR) was performed with gene-specific primers and iTaq Universal SYBR Green Supermix (Cat# 1725124, BioRad, Hercules, CA 94547, USA). Results were calculated using the –ΔΔCt method and a housekeeping gene (ACTB) as an internal control. The qPCR primers were mouse *Tnf* forward: GGTGCCTATGTCTCAGCCTCTT and reverse: GCCATAGAACTGATGAGAGGGAG, mouse *Casp11* forward: GTGGTGAAAGAGGAGCTTACAGC and reverse: GCACCAGGAATGTGCTGTCTGA, human *GBP1* forward: TCAATGAGGAAATCCCAGCCC and reverse: AGGCTGTTCCCTTGTCTGTTC, human *GBP2* forward: ATCTCTGATCTGGGGAACAACAC and reverse: GATAGAGGCCCACAATCGCC, human *GBP3* forward: AGCACAGACAAGAGAACAATGCC and reverse: TCTGGATTCGCCACCAGTTC, human *GBP4* forward: CAGTGCCCACACCAGGTTATC and reverse: TTCCTGTGCGGTATAGCCCT, human *GBP5* forward: CGGCGATTCAAAGGCAGAAC and reverse: AGCCTGTTCCTGCATCTGTTG, human *GBP6* forward: ACTGCACCATCCCATTTGTGG and reverse: TGCCAACCTAGAAGAGCCTGC, human *GBP7* forward: ACTCTGGACAGAGGAACGCC and reverse: TAGAGGCCCACAATTGCCAC.

### Isolation of splenic macrophages

Splenic macrophages were positively selected with biotin-F4/80 antibody and streptavidin nanobeads according to the manufacturer’s protocol (Cat# 480170, BioLegend). Briefly, spleens were finely minced on ice and resuspended in ice-cold MojoSort^™^ Buffer (4 mL/ spleen). The suspension was filtered through a 70 μm cell strainer, centrifuged at 300 g for 5 min, and diluted with MojoSort^™^ Buffer to 1 x 10^8^ cells/mL. The cell suspension was incubated with 4 μL of the biotin antibody cocktail per 10^7^ cells on ice for 15 min. MojoSort^™^ Buffer was added to the cell suspension up to 4 mL and centrifuged at 300 g for 5 min at 4°C (washing). The cell pellet was resuspended in 100 μL of MojoSort^™^ Buffer and 10 μL of nanobeads per 10^8^ cells. The mix was incubated on ice for 15 min and the cells/beads were washed as above. The cell/bead pellet was resuspended in 2.5 mL of MojoSort^™^ Buffer and placed in a magnet separator for 5 min on ice; and the unbound cells and liquid were removed (separation). This was repeated twice. The labeled cells were then used for RNA extraction.

### RNA sequencing

Total RNA was extracted from ~ 1 × 10^6^ cells using the RNAeasy Mini Plus kit (Qiagen Cat.# 74134). cDNA libraries were made with the VAHTS Universal V10 RNA-seq Library Prep Kit for Illumina. The libraries were quantified by Qubit and qPCR; fragment sizes were analyzed Qsep. The libraries were sequenced on the Element Biosciences AVITI platform with a 2x150 cycle sequencing kit. The quality of raw sequencing reads was assessed using FastQC. High-quality sequencing reads (>16.4 million per sample) were aligned to the mouse reference genome, mm39.fa mm39, and annotated with vM34.annotation.gtf vM34. Alignment and gene-level quantification were performed using STAR aligner. Differential expression analysis was conducted using DESeq2. Bioinformatics analyses were performed using Reactom.

### Graphing and statistical analyses

A Graphpad Prism10 software was used for graphing and statistical analyses. The sample sizes for animal experiments were estimated according to power analysis and our prior experience in similar experiments. Survival curves were analyzed using a log-rank (Mantel–Cox) test. A two-tailed, unpaired Student’s *t* test or non–parametric/parametric Mann–Whitney U test was applied to a dataset depending on its data distribution. The one-way or two-way ANOVA test was applied to comparing multiple groups simultaneously. *p* values ≤0.05 were considered significant. The sample sizes, statistical tests, and *p* values were specified in figure legends.

## Supplementary Material

This is a list of supplementary files associated with this preprint. Click to download.

- SupplementalFigS.pdf
- SupplementalTableS1RNAseqsourcedata.xlsx
- SupplementalvideoIMG6729.mov

## Data Availability

All the data generated or analyzed during this study are included in this published article (and its supplemental information files). The raw RNA-seq data are publicly available at https://www.ncbi.nlm.nih.gov/geo/, GSE306549. All the unique materials used are readily available from the authors. However, the availability of live animals may change over time.

## References

[1] Cahoon J., Yang D., and Wang P. (2022). The noncanonical inflammasome in health and disease. Infectious Medicine 1, 208–216. 38077630 10.1016/j.imj.2022.09.001 PMC10699704

[2] Rathinam V.A., and Fitzgerald K.A. (2016). Inflammasome Complexes: Emerging Mechanisms and Effector Functions. Cell 165, 792–800. 10.1016/j.cell.2016.03.046. 27153493 PMC5503689

[3] Downs K.P., Nguyen H., Dorfleutner A., and Stehlik C. (2020). An overview of the non-canonical inflammasome. Mol Aspects Med 76, 100924 10.1016/j.mam.2020.100924. 33187725 PMC7808250

[4] Kelley N., Jeltema D., Duan Y., and He Y. (2019). The NLRP3 Inflammasome: An Overview of Mechanisms of Activation and Regulation. Int J Mol Sci 20. 10.3390/ijms20133328. PMC6651423 31284572

[5] Man S.M., Karki R., Briard B., Burton A., Gingras S., Pelletier S., and Kanneganti T.D. (2017). Differential roles of caspase-1 and caspase-11 in infection and inflammation. Sci Rep 7, 45126. 10.1038/srep45126. 28345580 PMC5366862

[6] Deng M., Tang Y., Li W., Wang X., Zhang R., Zhang X., Zhao X., Liu J., Tang C., Liu Z., (2018). The Endotoxin Delivery Protein HMGB1 Mediates Caspase-11-Dependent Lethality in Sepsis. Immunity 49, 740–753 e747. 10.1016/j.immuni.2018.08.016. 30314759 PMC6300139

[7] Russo A.J., Vasudevan S.O., Mendez-Huergo S.P., Kumari P., Menoret A., Duduskar S., Wang C., Perez Saez J.M., Fettis M.M., Li C., (2021). Intracellular immune sensing promotes inflammation via gasdermin D-driven release of a lectin alarmin. Nat Immunol 22, 154–165. 10.1038/s41590-020-00844-7. 33398185 PMC8916041

[8] Tang Y., Zhang R., Xue Q., Meng R., Wang X., Yang Y., Xie L., Xiao X., Billiar T.R., and Lu B. (2018). TRIF signaling is required for caspase-11-dependent immune responses and lethality in sepsis. Mol Med 24, 66. 10.1186/s10020-018-0065-y. 30587103 PMC6307235

[9] Tang D., Wang H., Billiar T.R., Kroemer G., and Kang R. (2021). Emerging mechanisms of immunocoagulation in sepsis and septic shock. Trends in Immunology 42, 508–522. 10.1016/j.it.2021.04.001. 33906793 PMC8436187

[10] Zhu C., Liang Y., Luo Y., and Ma X. (2023). Role of pyroptosis in hemostasis activation in sepsis. Frontiers in Immunology 14. 10.3389/fimmu.2023.1114917. PMC9899792 36756123

[11] Ghait M., Duduskar S.N., Rooney M., Häfner N., Reng L., Göhrig B., Reuken P.A., Bloos F., Bauer M., Sponholz C., (2023). The non-canonical inflammasome activators Caspase-4 and Caspase-5 are differentially regulated during immunosuppression-associated organ damage. Frontiers in Immunology 14. 10.3389/fimmu.2023.1239474. PMC10722270 38106412

[12] Xu J., and Nunez G. (2022). The NLRP3 inflammasome: activation and regulation. Trends Biochem Sci. 10.1016/j.tibs.2022.10.002. PMC10023278 36336552

[13] Han S., Lear T.B., Jerome J.A., Rajbhandari S., Snavely C.A., Gulick D.L., Gibson K.F., Zou C., Chen B.B., and Mallampalli R.K. (2015). Lipopolysaccharide Primes the NALP3 Inflammasome by Inhibiting Its Ubiquitination and Degradation Mediated by the SCFFBXL2 E3 Ligase. J Biol Chem 290, 18124–18133. 10.1074/jbc.M115.645549. 26037928 PMC4505057

[14] Song H., Liu B., Huai W., Yu Z., Wang W., Zhao J., Han L., Jiang G., Zhang L., Gao C., and Zhao W. (2016). The E3 ubiquitin ligase TRIM31 attenuates NLRP3 inflammasome activation by promoting proteasomal degradation of NLRP3. Nat Commun 7, 13727. 10.1038/ncomms13727. 27929086 PMC5155141

[15] Wang D., Zhang Y., Xu X., Wu J., Peng Y., Li J., Luo R., Huang L., Liu L., Yu S., (2021). YAP promotes the activation of NLRP3 inflammasome via blocking K27-linked polyubiquitination of NLRP3. Nat Commun 12, 2674. 10.1038/s41467-021-22987-3. 33976226 PMC8113592

[16] Tang J., Tu S., Lin G., Guo H., Yan C., Liu Q., Huang L., Tang N., Xiao Y., Pope R.M., (2020). Sequential ubiquitination of NLRP3 by RNF125 and Cbl-b limits inflammasome activation and endotoxemia. J Exp Med 217. 10.1084/jem.20182091. PMC7144527 31999304

[17] Yan Y., Jiang W., Liu L., Wang X., Ding C., Tian Z., and Zhou R. (2015). Dopamine controls systemic inflammation through inhibition of NLRP3 inflammasome. Cell 160, 62–73. 10.1016/j.cell.2014.11.047. 25594175

[18] Humphries F., Bergin R., Jackson R., Delagic N., Wang B., Yang S., Dubois A.V., Ingram R.J., and Moynagh P.N. (2018). The E3 ubiquitin ligase Pellino2 mediates priming of the NLRP3 inflammasome. Nat Commun 9, 1560. 10.1038/s41467-018-03669-z. 29674674 PMC5908787

[19] Ni J., Guan C., Liu H., Huang X., Yue J., Xiang H., Jiang Z., Tao Y., Cao W., Liu J., (2021). Ubc13 Promotes K63-Linked Polyubiquitination of NLRP3 to Activate Inflammasome. J Immunol 206, 2376–2385. 10.4049/jimmunol.2001178. 33893171

[20] Labbe K., McIntire C.R., Doiron K., Leblanc P.M., and Saleh M. (2011). Cellular inhibitors of apoptosis proteins cIAP1 and cIAP2 are required for efficient caspase-1 activation by the inflammasome. Immunity 35, 897–907. 10.1016/j.immuni.2011.10.016. 22195745

[21] Nozaki K., Li L., and Miao E.A. (2022). Innate Sensors Trigger Regulated Cell Death to Combat Intracellular Infection. Annual Review of Immunology 40, 469–498. 10.1146/annurev-immunol-101320-011235. PMC9614550 35138947

[22] Liu Q., Zhang S., Sun Z., Guo X., and Zhou H. (2019). E3 ubiquitin ligase Nedd4 is a key negative regulator for non-canonical inflammasome activation. Cell Death Differ 26, 2386–2399. 10.1038/s41418-019-0308-7. 30816303 PMC6888843

[23] Geng T., Yang D., Lin T., Cahoon J.G., and Wang P. (2022). UBXN3B Controls Immunopathogenesis of Arthritogenic Alphaviruses by Maintaining Hematopoietic Homeostasis. mBio 13, e0268722. 10.1128/mbio.02687-22. 36377866 PMC9765034

[24] Geng T., Yang D., Lin T., Harrison A.G., Wang B., Cao Z., Torrance B., Fan Z., Wang K., Wang Y., (2024). UBXN3B is crucial for B lymphopoiesis. EBioMedicine 106, 105248. 10.1016/j.ebiom.2024.105248. 39018756 PMC11287013

[25] Yang L., Wang L., Ketkar H., Ma J., Yang G., Cui S., Geng T., Mordue D.G., Fujimoto T., Cheng G., (2018). UBXN3B positively regulates STING-mediated antiviral immune responses. Nat Commun 9, 2329. 10.1038/s41467-018-04759-8. 29899553 PMC5998066

[26] Hu Y., O'Boyle K., Auer J., Raju S., You F., Wang P., Fikrig E., and Sutton R.E. (2017). Multiple UBXN family members inhibit retrovirus and lentivirus production and canonical NFkappaBeta signaling by stabilizing IkappaBalpha. PLoS Pathog 13, e1006187. 10.1371/journal.ppat.1006187. 28152074 PMC5308826

[27] Kayagaki N., Wong M.T., Stowe I.B., Ramani S.R., Gonzalez L.C., Akashi-Takamura S., Miyake K., Zhang J., Lee W.P., Muszynski A., (2013). Noncanonical inflammasome activation by intracellular LPS independent of TLR4. Science 341, 1246–1249. 10.1126/science.1240248. 23887873

[28] Dejager L., Pinheiro I., Dejonckheere E., and Libert C. (2011). Cecal ligation and puncture: the gold standard model for polymicrobial sepsis? Trends Microbiol 19, 198–208. 10.1016/j.tim.2011.01.001. 21296575

[29] Wesa A.K., and Galy A. (2001). IL-1β induces dendritic cells to produce IL-12. International Immunology 13, 1053–1061. 10.1093/intimm/13.8.1053. 11470775

[30] Weber A., Wasiliew P., and Kracht M. (2010). Interleukin-1 (IL-1) pathway. Sci Signal 3, cm1. 10.1126/scisignal.3105cm1. 20086235

[31] Rex D.A.B., Agarwal N., Prasad T.S.K., Kandasamy R.K., Subbannayya Y., and Pinto S.M. (2020). A comprehensive pathway map of IL-18-mediated signalling. J Cell Commun Signal 14, 257–266. 10.1007/s12079-019-00544-4. 31863285 PMC7272533

[32] Alexandru G., Graumann J., Smith G.T., Kolawa N.J., Fang R., and Deshaies R.J. (2008). UBXD7 binds multiple ubiquitin ligases and implicates p97 in HIF1alpha turnover. Cell 134, 804–816. 10.1016/j.cell.2008.06.048. 18775313 PMC2614663

[33] Dikic I., Wakatsuki S., and Walters K.J. (2009). Ubiquitin-binding domains - from structures to functions. Nat Rev Mol Cell Biol 10, 659–671. 10.1038/nrm2767. 19773779 PMC7359374

[34] Lange S.M., McFarland M.R., Lamoliatte F., Carroll T., Krshnan L., Perez-Rafols A., Kwasna D., Shen L., Wallace I., Cole I., (2024). VCP/p97-associated proteins are binders and debranching enzymes of K48-K63-branched ubiquitin chains. Nat Struct Mol Biol. 10.1038/s41594-024-01354-y. PMC11638074 38977901

[35] Blount J.R., Johnson S.L., and Todi S.V. (2020). Unanchored Ubiquitin Chains, Revisited. Front Cell Dev Biol 8, 582361. 10.3389/fcell.2020.582361. 33195227 PMC7659471

[36] Otten E.G., Werner E., Crespillo-Casado A., Boyle K.B., Dharamdasani V., Pathe C., Santhanam B., and Randow F. (2021). Ubiquitylation of lipopolysaccharide by RNF213 during bacterial infection. Nature 594, 111–116. 10.1038/s41586-021-03566-4. 34012115 PMC7610904

[37] Shi J., Zhao Y., Wang Y., Gao W., Ding J., Li P., Hu L., and Shao F. (2014). Inflammatory caspases are innate immune receptors for intracellular LPS. Nature 514, 187–192. 10.1038/nature13683. 25119034

[38] Dickinson M.S., Kutsch M., Sistemich L., Hernandez D., Piro A.S., Needham D., Lesser C.F., Herrmann C., and Coers J. (2023). LPS-aggregating proteins GBP1 and GBP2 are each sufficient to enhance caspase-4 activation both in cellulo and in vitro. Proc Natl Acad Sci U S A 120, e2216028120. 10.1073/pnas.2216028120. 37023136 PMC10104521

[39] Fisch D., Bando H., Clough B., Hornung V., Yamamoto M., Shenoy A.R., and Frickel E.M. (2019). Human GBP1 is a microbe-specific gatekeeper of macrophage apoptosis and pyroptosis. EMBO J 38, e100926. 10.15252/embj.2018100926. 31268602 PMC6600649

[40] Santos J.C., Boucher D., Schneider L.K., Demarco B., Dilucca M., Shkarina K., Heilig R., Chen K.W., Lim R.Y.H., and Broz P. (2020). Human GBP1 binds LPS to initiate assembly of a caspase-4 activating platform on cytosolic bacteria. Nat Commun 11, 3276. 10.1038/s41467-020-16889-z. 32581219 PMC7314798

[41] Wandel M.P., Kim B.H., Park E.S., Boyle K.B., Nayak K., Lagrange B., Herod A., Henry T., Zilbauer M., Rohde J., (2020). Guanylate-binding proteins convert cytosolic bacteria into caspase-4 signaling platforms. Nat Immunol 21, 880–891. 10.1038/s41590-020-0697-2. 32541830 PMC7381384

[42] Wang P., Yang L., Cheng G., Yang G., Xu Z., You F., Sun Q., Lin R., Fikrig E., and Sutton R.E. (2013). UBXN1 interferes with Rig-I-like receptor-mediated antiviral immune response by targeting MAVS. Cell Rep 3, 1057–1070. 10.1016/j.celrep.2013.02.027. 23545497 PMC3707122

[43] Wang Y.B., Tan B., Mu R., Chang Y., Wu M., Tu H.Q., Zhang Y.C., Guo S.S., Qin X.H., Li T., (2015). Ubiquitin-associated domain-containing ubiquitin regulatory X (UBX) protein UBXN1 is a negative regulator of nuclear factor kappaB (NF-kappaB) signaling. J Biol Chem 290, 10395–10405. 10.1074/jbc.M114.631689. 25681446 PMC4400349

[44] Ganji R., Mukkavalli S., Somanji F., and Raman M. (2018). The VCP-UBXN1 Complex Mediates Triage of Ubiquitylated Cytosolic Proteins Bound to the BAG6 Complex. Mol Cell Biol 38. 10.1128/MCB.00154-18. PMC6002697 29685906

[45] Ishibashi T., Ogawa S., Hashiguchi Y., Inoue Y., Udo H., Ohzono H., Kato A., Minakami R., and Sugiyama H. (2005). A novel protein specifically interacting with Homer2 regulates ubiquitin-proteasome systems. J Biochem 137, 617–623. 10.1093/jb/mvi074. 15944415

[46] LaLonde D.P., and Bretscher A. (2011). The UBX protein SAKS1 negatively regulates endoplasmic reticulum-associated degradation and p97-dependent degradation. J Biol Chem 286, 4892–4901. 10.1074/jbc.M110.158030. 21135095 PMC3039385

[47] Park E.S., Yoo Y.J., and Elangovan M. (2017). The opposite role of two UBA-UBX containing proteins, p47 and SAKS1 in the degradation of a single ERAD substrate, alpha-TCR. Mol Cell Biochem 425, 37–45. 10.1007/s11010-016-2860-5. 27785701

[48] Shahul Hameed D., van Tilburg G.B.A., Merkx R., Flierman D., Wienk H., El Oualid F., Hofmann K., Boelens R., and Ovaa H. (2019). Diubiquitin-Based NMR Analysis: Interactions Between Lys6-Linked diUb and UBA Domain of UBXN1. Front Chem 7, 921. 10.3389/fchem.2019.00921. 32039147 PMC6987245

[49] Song Y.K., Hu B.C., Xu L., Liu J.Q., Chen X., Zheng Y., Chen M.H., Wang J.Z., Sun R.H., and Mo S.J. (2019). Productive transcription of miR-124-3p by RelA and RNA polymerase II directs RIP1 ubiquitination-dependent apoptosis resistance during hypoxia. Exp Cell Res 378, 21–31. 10.1016/j.yexcr.2019.03.004. 30844390

[50] Wu-Baer F., Ludwig T., and Baer R. (2010). The UBXN1 protein associates with autoubiquitinated forms of the BRCA1 tumor suppressor and inhibits its enzymatic function. Mol Cell Biol 30, 2787–2798. 10.1128/MCB.01056-09. 20351172 PMC2876507

[51] Mukkavalli S., Klickstein J.A., Ortiz B., Juo P., and Raman M. (2021). The p97-UBXN1 complex regulates aggresome formation. J Cell Sci 134. 10.1242/jcs.254201. PMC8077447 33712450

[52] Mengus C., Neutzner M., Bento A., Bippes C.C., Kohler C., Decembrini S., Hausel J., Hemion C., Sironi L., Frank S., (2021). VCP/p97 cofactor UBXN1/SAKS1 regulates mitophagy by modulating MFN2 removal from mitochondria. Autophagy, 1–20. 10.1080/15548627.2021.1922982. PMC8865314 33966597

[53] Ma L., Han T., and Zhan Y.A. (2024). Mechanism and role of mitophagy in the development of severe infection. Cell Death Discov 10, 88. 10.1038/s41420-024-01844-4. 38374038 PMC10876966

[54] Liu S.F., and Malik A.B. (2006). NF-kappa B activation as a pathological mechanism of septic shock and inflammation. Am J Physiol Lung Cell Mol Physiol 290, L622–L645. 10.1152/ajplung.00477.2005. 16531564

[55] Amerik A., Swaminathan S., Krantz B.A., Wilkinson K.D., and Hochstrasser M. (1997). In vivo disassembly of free polyubiquitin chains by yeast Ubp14 modulates rates of protein degradation by the proteasome. EMBO J 16, 4826–4838. 10.1093/emboj/16.16.4826. 9305625 PMC1170118

[56] Dayal S., Sparks A., Jacob J., Allende-Vega N., Lane D.P., and Saville M.K. (2009). Suppression of the deubiquitinating enzyme USP5 causes the accumulation of unanchored polyubiquitin and the activation of p53. J Biol Chem 284, 5030–5041. 10.1074/jbc.M805871200. 19098288 PMC2696100

[57] Hadari T., Warms J.V., Rose I.A., and Hershko A. (1992). A ubiquitin C-terminal isopeptidase that acts on polyubiquitin chains. Role in protein degradation. J Biol Chem 267, 719–727. 1309773

[58] Piotrowski J., Beal R., Hoffman L., Wilkinson K.D., Cohen R.E., and Pickart C.M. (1997). Inhibition of the 26 S proteasome by polyubiquitin chains synthesized to have defined lengths. J Biol Chem 272, 23712–23721. 10.1074/jbc.272.38.23712. 9295315

[59] Thrower J.S., Hoffman L., Rechsteiner M., and Pickart C.M. (2000). Recognition of the polyubiquitin proteolytic signal. EMBO J 19, 94–102. 10.1093/emboj/19.1.94. 10619848 PMC1171781

[60] Xia Z.P., Sun L., Chen X., Pineda G., Jiang X., Adhikari A., Zeng W., and Chen Z.J. (2009). Direct activation of protein kinases by unanchored polyubiquitin chains. Nature 461, 114–119. 10.1038/nature08247. 19675569 PMC2747300

[61] Catici D.A., Horne J.E., Cooper G.E., and Pudney C.R. (2015). Polyubiquitin Drives the Molecular Interactions of the NF-kappaB Essential Modulator (NEMO) by Allosteric Regulation. J Biol Chem 290, 14130–14139. 10.1074/jbc.M115.640417. 25866210 PMC4447983

[62] Rahighi S., Ikeda F., Kawasaki M., Akutsu M., Suzuki N., Kato R., Kensche T., Uejima T., Bloor S., Komander D., (2009). Specific recognition of linear ubiquitin chains by NEMO is important for NF-kappaB activation. Cell 136, 1098–1109. 10.1016/j.cell.2009.03.007. 19303852

[63] Skaug B., Chen J., Du F., He J., Ma A., and Chen Z.J. (2011). Direct, noncatalytic mechanism of IKK inhibition by A20. Mol Cell 44, 559–571. 10.1016/j.molcel.2011.09.015. 22099304 PMC3237303

[64] Zeng W., Sun L., Jiang X., Chen X., Hou F., Adhikari A., Xu M., and Chen Z.J. (2010). Reconstitution of the RIG-I pathway reveals a signaling role of unanchored polyubiquitin chains in innate immunity. Cell 141, 315–330. 10.1016/j.cell.2010.03.029. 20403326 PMC2919214

[65] Jiang X., Kinch L.N., Brautigam C.A., Chen X., Du F., Grishin N.V., and Chen Z.J. (2012). Ubiquitin-induced oligomerization of the RNA sensors RIG-I and MDA5 activates antiviral innate immune response. Immunity 36, 959–973. 10.1016/j.immuni.2012.03.022. 22705106 PMC3412146

[66] Rajsbaum R., Versteeg G.A., Schmid S., Maestre A.M., Belicha-Villanueva A., Martínez-Romero C., Patel J.R., Morrison J., Pisanelli G., Miorin L., (2014). Unanchored K48-linked polyubiquitin synthesized by the E3-ubiquitin ligase TRIM6 stimulates the interferon-IKKε kinase-mediated antiviral response. Immunity 40, 880–895. 10.1016/j.immuni.2014.04.018. 24882218 PMC4114019

[67] Herwanto V., Tang B., Wang Y., Shojaei M., Nalos M., Shetty A., Lai K., McLean A.S., and Schughart K. (2021). Blood transcriptome analysis of patients with uncomplicated bacterial infection and sepsis. BMC Research Notes 14, 76. 10.1186/s13104-021-05488-w. 33640018 PMC7913415

[68] Kayagaki N., Warming S., Lamkanfi M., Vande Walle L., Louie S., Dong J., Newton K., Qu Y., Liu J., Heldens S., (2011). Non-canonical inflammasome activation targets caspase-11. Nature 479, 117–121. 10.1038/nature10558. 22002608

[69] Nakhaei P., Mesplede T., Solis M., Sun Q., Zhao T., Yang L., Chuang T.-H., Ware C.F., Lin R., and Hiscott J. (2009). The E3 Ubiquitin Ligase Triad3A Negatively Regulates the RIG-I/MAVS Signaling Pathway by Targeting TRAF3 for Degradation. PLOS Pathogens 5, e1000650. 10.1371/journal.ppat.1000650. 19893624 PMC2766052

[70] Shivcharan S., Younis D.A., Wright S.S., Wang C., Behl B., Shumba P., Delgado K.N., Chawla A.K., Alto N.M., Panjwani N., (2025). Endolysosomal damage surveillance enables rapid inflammasome sensing of pathogens. Cell Rep 44, 116002. 10.1016/j.celrep.2025.116002. 40668675 PMC12871383

[71] Li J., Brieher W.M., Scimone M.L., Kang S.J., Zhu H., Yin H., von Andrian U.H., Mitchison T., and Yuan J. (2007). Caspase-11 regulates cell migration by promoting Aip1-Cofilin-mediated actin depolymerization. Nat Cell Biol 9, 276–286. 10.1038/ncb1541. 17293856

[72] Stennicke H.R., and Salvesen G.S. (1997). Biochemical characteristics of caspases-3, −6, −7, and −8. J Biol Chem 272, 25719–25723. 10.1074/jbc.272.41.25719. 9325297

[73] Xu Y., Zhang X., Tang X., Zhang C., Cahoon J.G., Wang Y., Li H., Lv X., Wang Y., Wang Z., (2024). Dexmedetomidine post-treatment exacerbates metabolic disturbances in septic cardiomyopathy via α(2A)-adrenoceptor. Biomed Pharmacother 170, 115993. 10.1016/j.biopha.2023.115993. 38091635

[74] Yang D., Dai X., Xing Y., Tang X., Yang G., Harrison A.G., Cahoon J., Li H., Lv X., Yu X., (2022). Intrinsic cardiac adrenergic cells contribute to LPS-induced myocardial dysfunction. Commun Biol 5, 96. 10.1038/s42003-022-03007-6. 35079095 PMC8789803

[75] Yang D., Geng T., Harrison A.G., Cahoon J.G., Xing J., Jiao B., Wang M., Cheng C., Hill R.E., Wang H., (2024). UBR5 promotes antiviral immunity by disengaging the transcriptional brake on RIG-I like receptors. Nature Communications 15, 780. 10.1038/s41467-024-45141-1. PMC10817939 38278841

[76] Sanjana N.E., Shalem O., and Zhang F. (2014). Improved vectors and genome-wide libraries for CRISPR screening. Nat Methods 11, 783–784. 10.1038/nmeth.3047. 25075903 PMC4486245

[77] Stewart S.A., Dykxhoorn D.M., Palliser D., Mizuno H., Yu E.Y., An D.S., Sabatini D.M., Chen I.S., Hahn W.C., Sharp P.A., (2003). Lentivirus-delivered stable gene silencing by RNAi in primary cells. RNA 9, 493–501. 10.1261/rna.2192803. 12649500 PMC1370415

[78] Yang D., Lin T., Li C., Harrison A.G., Geng T., and Wang P. (2021). A critical role for MSR1 in vesicular stomatitis virus infection of the central nervous system. iScience 24, 102678. 10.1016/j.isci.2021.102678. 34169243 PMC8208900

[79] Yang D.M., Geng T.T., Harrison A.G., and Wang P.H. (2021). Differential roles of RIG-I like receptors in SARS-CoV-2 infection. Mil Med Res 8, 49. 10.1186/s40779-021-00340-5. 34488908 PMC8421188

